# An advanced comprehensive muti-cell-type-specific model for predicting anti-PD-1 therapeutic effect in melanoma

**DOI:** 10.7150/thno.91626

**Published:** 2024-03-03

**Authors:** Wei Sun, Yu Zhu, Zijian Zou, Lu Wang, Jingqin Zhong, Kangjie Shen, Xinyi Lin, Zixu Gao, Wanlin Liu, Yinlam Li, Yu Xu, Ming Ren, Tu Hu, Chuanyuan Wei, Jianying Gu, Yong Chen

**Affiliations:** 1 Department of Musculoskeletal Oncology, Fudan University Shanghai Cancer Center; Department of Oncology, Shanghai Medical College, Fudan University, Shanghai 200032, P. R. China.; 2 Department of Plastic and Reconstructive Surgery, Zhongshan Hospital, Fudan University; Cancer center, Zhongshan Hospital, Fudan University, Shanghai 200032, P. R. China.

**Keywords:** anti-PD-1, immune checkpoint inhibitors, machine learning, malignant melanoma, tumor microenvironment

## Abstract

**Rationale:** Immune checkpoint inhibitors targeting the programmed cell death (PD)-1/PD-L1 pathway have promise in patients with advanced melanoma. However, drug resistance usually results in limited patient benefits. Recent single-cell RNA sequencing studies have elucidated that MM patients display distinctive transcriptional features of tumor cells, immune cells and interstitial cells, including loss of antigen presentation function of tumor cells, exhaustion of CD8+T and extracellular matrix secreted by fibroblasts to prevents immune infiltration, which leads to a poor response to immune checkpoint inhibitors (ICIs). However, cell subgroups beneficial to anti-tumor immunity and the model developed by them remain to be further identified.

**Methods:** In this clinical study of neoadjuvant therapy with anti-PD-1 in advanced melanoma, tumor tissues were collected before and after treatment for single-nucleus sequencing, and the results were verified using multicolor immunofluorescence staining and public datasets.

**Results:** This study describes four cell subgroups which are closely associated with the effectiveness of anti-PD-1 treatment. It also describes a cell-cell communication network, in which the interaction of the four cell subgroups contributes to anti-tumor immunity. Furthermore, we discuss a newly developed predictive model based on these four subgroups that holds significant potential for assessing the efficacy of anti-PD-1 treatment.

**Conclusions:** These findings elucidate the primary mechanism of anti-PD-1 resistance and offer guidance for clinical drug administration for melanoma.

## Introduction

Malignant melanoma (MM) is a kind of malignancy arising from the transformation of melanocytes and is acknowledged as one of the most lethal tumors worldwide. Despite a global decline in the incidence of several cancer types, the occurrence of melanoma is on the rise 1, 2. CD8^+^ T cells play a crucial role in anti-tumor immunity through direct contact-mediated cytotoxicity and are integral to cellular immunity. PD-1, expressed on the surface of T cells, induces T cell dysfunction, exhaustion and apoptosis when stimulated by PD-L1. This inhibits the activation, proliferation, and anti-tumor function of CD8^+^ T cells specific to tumor antigens, leading to tumor immune evasion 3, 4. Anti-PD-1 (PD-1 antibody) binds to CD8^+^ T cells, preventing the interaction between PD-1 and its ligands, thereby restoring suppressed tumor-specific T cell cytotoxicity 5. Immune checkpoint inhibitors (ICIs), with anti-PD-1 being a representative example, have significantly improved the previously poor prognosis of MM 6. However, despite the excellent efficacy of anti-PD-1 in melanoma compared to other tumors, many patients still develop primary and secondary drug resistance, resulting in a low objective response rate (ORR) ranging from 16.6% to 38% 7-10. Therefore, the identification of advanced biomarkers is crucial for predicting the efficacy of ICIs treatment and improving drug selection strategies to overcome intrinsic drug resistance.

The emergence of single-nucleus sequencing (snRNA-seq) has provided a novel research perspective, allowing us to examine gene expression patterns in the cellular stratum and the distinctive characteristics enabled by snRNA-seq, thereby enhancing our ability to predict prognosis 11-13. Previous studies have demonstrated that various cell types within the tumor microenvironment (TME) exert different effects on the prognosis of ICIs treatment. Livnat et al. revealed that melanoma cells induce immunosuppression through autonomous programs associated with T cell exclusion 14. Similarly, He et al. identified tumor antigen-specific T cells with elevated expression of C-X-C motif chemokine ligand (CXCL) 13 as potential prognostic biomarkers for ICIs treatment 15. B cells, myeloid cells, fibroblasts, and endothelial cells in the TME have been associated with the prognosis of ICIs treatment, leading to the development of various models for predicting treatment response 16-19. However, due to the complexity of the TME, a single cell subgroup alone cannot account for all influencing factors, necessitating a refined approach to construct a prognostic model based on the single-cell atlas.

In this study, we conducted a comprehensive analysis of snRNA-seq data from MM samples before and after anti-PD-1 treatment with varying therapeutic effects. Our findings demonstrate that the TME of the PD-pre group exhibited high suppression, which was reversed by anti-PD-1 treatment, leading to an increase in CD8^+^ T cells. We identified subgroups of tumor cells, lymphocytes, myeloid cells, and stromal cells that were either diminished or absent in the PD-pre group. These four subgroups were found to possess distinct immune activation functions that promote tumor immunity through cell interactions. Furthermore, using seven machine learning algorithms and available pre-treatment Bulk-RNA data from MM patients treated with anti-PD-1, we developed 35 models. Among these models, the AdaBoost model constructed from the combined gene set demonstrated superior predictive capability and accuracy for anti-PD-1 treatment compared to previously reported models. Our study elucidates the primary mechanism of anti-PD-1 resistance and provides guidance for clinical drug administration and combination therapy for melanoma.

## Results

### Single-cell transcriptome atlas of melanoma patients with neoadjuvant anti-PD-1 therapy

The study was divided into four stages: diagnosis, treatment, surgery, and follow-up **(Figure S1A)**. To confirm the malignancy and tumor stage of MM patients, imaging examination (enhanced computed tomography (CT)) and histopathological examination (needle biopsy) were performed. Patients with definite stage III-IV malignant melanoma received two courses of toripalimab (240 mg once every two weeks for a total duration of four weeks). After five weeks, enhanced CT was performed to evaluate the treatment response, dividing patients into the PD (progressive disease) and NPD (non-progressive disease) groups based on the curative effect** (Figure S1B)**. Patients in the NPD group underwent radical surgical resection. Representative hematoxylin and eosin (H&E)-stained images of tumors are shown in **Figure S1C**, illustrating the natural tumor microenvironment (TME) before treatment (left two panels) and tumor necrosis (upper right panel) and fibrosis after treatment (lower right panel). We collected data from 11 melanoma patients who received neoadjuvant anti-PD-1 therapy, including three patients in the PD group and eight patients in the NPD group **(Table S1)**. Additional information about these patients is presented in **Table S2**. Paired pre-treatment (NPD-pre) and post-treatment (NPD-post) tumor tissues were collected from patients in the NPD group, while only pre-treatment (PD-pre) tumor tissues were collected from patients in the PD group.

**Figure 1:**
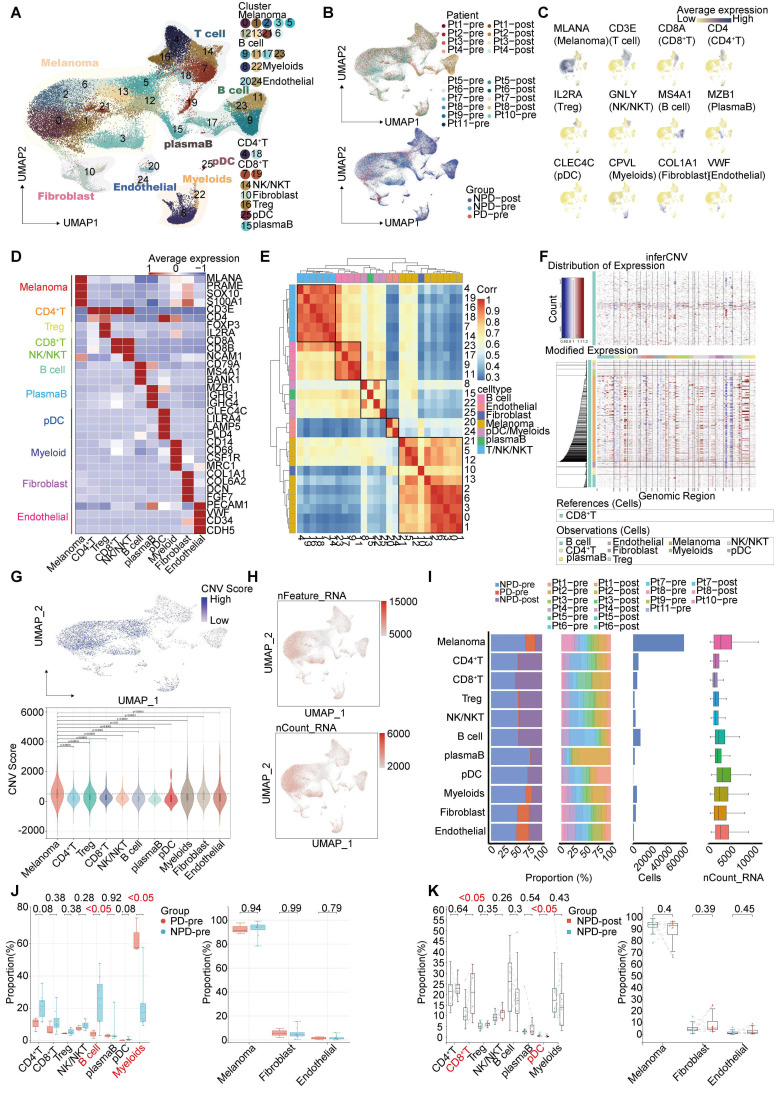
** Single-nucleus profiles of the melanoma ecosystem. A.** UMAP plot of 100 554 cells from primary tumor tissues of 11 melanoma patients including pre-treatment and post-treatment, showing the annotation and color codes for cell types in the melanoma ecosystem. **B.** UMAP plot indicating the patient origin and group for each single cell. **C.** Feature plots showing classical marker genes for the annotated 11 cell types. **D.** Heatmap showing the expression of selected marker genes in the annotated cell types. **E.** Heatmap depicts pairwise correlations of 26 clusters derived from 11 patients. Clustering identifies five coherent expression programs across tumors. **F.** Hierarchical heatmap showing large-scale CNVs in 11 subgroups. CD8^+^T is the reference subgroup. **G.** Feature plots for CNV score; Violin plots showing CNV scores cross 11 subgroups (non-paired t-test). **H.** Feature plots for nFeature_RNA (the number of genes detected) and nCount_RNA (the number of mRNA molecules detected). **I.** Bar plots showing the fraction of cells originating from NPD-pre, PD-pre and NPD-post group, the fraction of cells originating from each of the patients, and the number of cells, and box plots showing the nCount_RNA of the 11 annotated cell types. **J.** Box plot showing the proportion of annotated 11 subgroups between PD-pre and NPD-pre groups (non-paired t test). K. Box plot showing the proportion of annotated 11 subgroups between NPD-pre and NPD-pre groups (paired t-test).

Single-cell suspensions were generated from all freshly frozen tumor tissues and analyzed using droplet-based single-cell transcriptome profiling. After quality filtering and doublet removal, a total of 100,554 single cells from melanoma samples were profiled. Principal component analysis was applied to evaluate variably expressed genes, and a graph-based clustering method was used to classify the cells into coherent transcriptional clusters. Cells from different patients and groups were well mixed** (Fig. 1A-B)**. Cell clusters were annotated based on the average expression of canonical marker genes, identifying melanoma cells, immune cells (such as myeloid cells, plasmacytoid dendritic cells (pDCs), CD8^+^ T cells, CD4^+^ T cells, regulatory T cells (Tregs), natural killer/natural killer T (NK/NKT) cells **(Figure S2)**, and B cells), fibroblasts, endothelial cells, and epithelial cells **(Figure 1C-D)**. Clusters belonging to the same cell type exhibited good correlation at the transcriptional level **(Figure 1E)**. The differentiation between malignant and non-malignant cells was validated using a two-step process. Firstly, the copy number variation (CNV) score of each cell was determined, and elevated CNV scores were observed in melanoma cells **(Figure 1F-G)**. Secondly, the number of expressed genes and total gene counts were also enriched in melanoma cells **(Figure 1H-I)**, confirming the accuracy of our cell types for subsequent analyses.

We compared the proportions of these cell types and found that the number of lymphocytes, including CD4^+^ T, CD8^+^ T, NK/NKT, and B cells, was lower in the PD-pre group compared to the NPD-pre group** (Figure 1I)**. In patients in the PD-pre group, the proportion of B cells significantly decreased (p < 0.05), while the proportion of myeloid cells significantly increased (p < 0.05) **(Figure 1J)**. Furthermore, we observed a significant increase (p < 0.05) in CD8^+^ T cells and a significant reduction (p < 0.05) in pDCs following anti-PD-1 neoadjuvant therapy **(Figure 1K)**. We also conducted a preliminary identification of common immune inhibitory cell subtypes, but no significant differences were observed between the PD-pre and NPD-pre groups **(Figure S3)**. These findings indicate that patients who are insensitive to anti-PD-1 therapy present with a suppressed TME.

### High heterogeneity of melanoma cells at single-cell level

**Figure 2:**
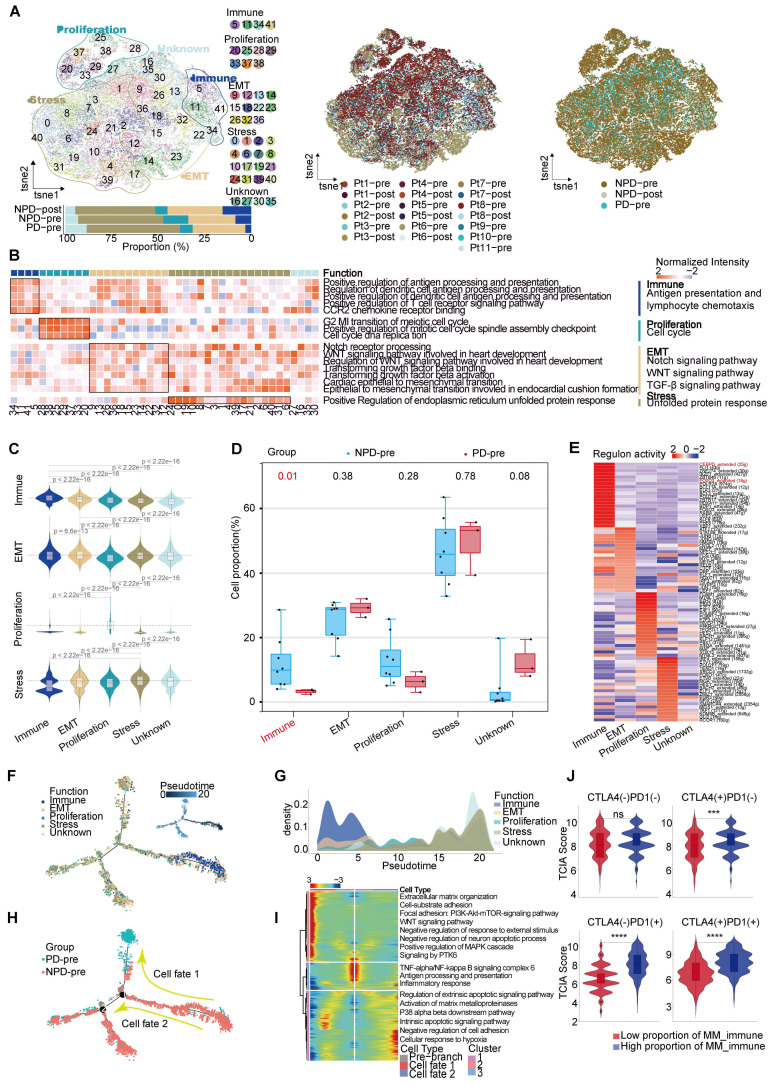
** Functions and pseudo-temporal trajectories of distinct melanoma cell subgroups. A.** t-SNE plot (upper panel) of 56 664 melanoma cells, color-coded by their associated cluster, patients, and groups; Bar plot (lower panel) showing the proportions of each subgroup. **B.** Heatmap showing the function score by GSVA in the 41 clusters (5 subgroup), including biological functions and names of related signal pathways. **C.** Heatmap showing the specifically highly expressed genes in each subgroup. **D.** Box plot showing the proportion of annotated melanoma subgroups between PD-pre and NPD-pre groups (non-paired t test). **E.** Heatmap of the t-values of AUC scores of expression regulation by transcription factors, as estimated using SCENIC, per annotated subgroup. **F.** Pseudo-temporal trajectory (big panel) of pre-treatment melanoma cells identified two distinct cell fates colored by subgroup; Pseudo-temporal trajectory (small panel) of pre-treatment melanoma cells identified two distinct cell fates colored by Pseudo-time. **G.** The cell density distribution of the pseudotime-ordered melanoma cells from NPD-pre and PD-pre groups. **H.** Pseudo-temporal trajectory of PD-pre and NPD-pre melanoma cells identified two distinct cell fates colored by groups. **I.** Heatmap showing the enriched pathways in these three phases of **H) J.** Violin plots of TCIA score of TCGA patients categorized into high and low groups based on their score of the percentage of the MM_immune subgroup (non-paired t test).

After conducting unsupervised reclustering of melanoma cells, a total of 56,664 melanoma cells were classified into 41 clusters, which were further divided into five primary subgroups based on their biological function determined by Gene Ontology (GO) analysis (see Methods). These five subgroups were labeled MM_Immune, MM_Epithelial-mesenchymal transition (EMT), MM_Proliferation, MM_Stress, and MM_Unknown (unable to be assigned to a specific functional group and thus classified as unknown) **(Figure 2A)**. Each subgroup exhibited unique functional signatures.The MM_Immune subgroup was involved in antigen presentation and lymphocyte chemotaxis. The MM_EMT subgroup was enriched in pathways associated with epithelial-mesenchymal transition, such as NOTCH, WNT, and TGF-β signaling pathways. The MM_Proliferation subgroup was associated with cell cycle circuits, and the MM_Stress subgroup was related to the unfolded protein response** (Figure 2B)**.

To validate the accuracy of the subgroup classification, differential gene expression and gene set variation analysis (GSVA) were performed **(Figure S4A-B)**. As expected, MM_Immune cells exhibited numerous genes related to antigen presentation and chemokines, including CD74 molecule (CD74), major histocompatibility complex, class II, DR alpha (HLA-DRA), immunoglobulin kappa constant (IGKC), and C-C motif chemokine ligand (CCL) 21 20-22. The MM_EMT subgroup exhibited elevated expression of genes associated with metastasis, such as vimentin (VIM), actin beta (ACTB), and secreted phosphoprotein 1 (SPP1) 23-25. The MM_Proliferative subgroup exhibited increased expression of genes related to the cell cycle, such as DNA topoisomerase II alpha (TOP2A) and enhancer of zeste 2 polycomb repressive complex 2 subunit (EZH2). This subgroup was most enriched for E2F transcription factor targets and G2M checkpoints pathway. Finally, the MM_Stress subgroup exhibited elevated expression of stress-related genes, including SRY (sex determining region Y)-box transcription factor 6 (SOX6), tyrosinase (TYR), nuclear factor I A (NFIA), and inner mitochondrial membrane peptidase subunit 2 (IMMP2L), which are related to the regulation of cellular responses to oxidative stress 26-29. The MM cell subgroup characterized by immune modulation, WNT signaling, and TGF-β signaling pathway activation features has been discovered in previous studies 30, 31. Furthermore, functional scores were assessed to validate the gene expression signatures of the subgroups. The highest scores were observed in the corresponding functions of the immune, EMT, proliferation, and stress subgroups **(Figure 2C)**, these results indicated a notable functional heterogeneity among MM subgroups.

### Decreased MM_Immune subgroup predicts insensitive to anti-PD-1 therapy

We observed a significantly higher cell proportion of the MM_Immune subgroup in the NPD-pre group compared to the PD-pre group (p < 0.05). However, there were no differences in the remaining subgroups between the NPD-pre and NPD-post groups **(Figure 2A & D, Figure S4C)**. In the t-SNE plot, genes associated with antigen presentation, namely CD74 and HLA-DRA, were clustered within the MM_Immune subgroup.

Additionally, immune-related genes such as IGKC and immunoglobulin heavy constant gamma 1 (*IGHG1*) were highly expressed in this subgroup, suggesting that the immune-driving capacity of the MM_Immune subgroup is influenced by immune-related genes **(Figure S4D)**. Based on these findings, we speculate that the MM_Immune subgroup plays a crucial role in determining the efficacy of anti-PD-1 therapy in melanoma.We then mapped the gene regulatory networks that govern these subgroups and found that MM_Immune cells exhibited elevated regulon activity for CCAAT enhancer binding protein delta (CEBPD) and forkhead box D3 (FOXD3) **(Figure 2E)**. The CEBPD/toll-like receptor 4 (TLR4) axis regulates inflammation, and the absence of the FOXD3/V-set immunoregulatory receptor (VISTA) axis results in increased expression of PD-L1, which may explain the immune activation status observed in the MM_Immune subgroup 32, 33. Further analysis confirmed that within the MM_Immune subgroup, the proportion of PD-L1-positive cells in tumor cells was the lowest, potentially due to the influence of FOXD3** (Figure S4E).** However, it is important to note that the proportion of PD-L1-positive cells is lower in the PD-pre group compared to the NPD-pre group, which may provide an explanation for the fact that the expression level of PD-L1 is not the sole determinant of anti-PD-1 therapy efficacy.

To obtain the MM pseudo-time trajectory, we randomly selected 11,000 high-quality pre-treatment tumor cells (including the PD-pre and NPD-pre groups) to establish a pseudo-temporal trajectory. MM_Immune cells were primarily situated at the beginning of the phylogenetic tree and gradually decreased during development **(Figure 2F)**. By generating a pseudo-time trajectory for melanoma cells in the NPD-pre and NPD-post groups, we confirmed that the MM_Immune subgroup was positioned at the root, while the MM_Stress subgroup was located at the terminus of the phylogenetic tree **(Figure S4F)**. These findings suggest that MM_Immune cells represent the initial tumor cells that undergo transformation and develop into a subgroup of cells with alternative functions. However, MM_Immune cells lose their antigen-presenting characteristics, which may contribute to the observed phenomenon of drug resistance in ICIs treatment **(Figure 2G)**. Furthermore, when examining the cell fate downstream of branching point 1, we observed that tumor cells in the PD-pre group mainly gathered in cell fate 1, whereas tumor cells in the NPD-pre group primarily gathered in the pre-branch and cell fate 2 **(Figure 2H)**. To investigate this phenomenon, we conducted gene expression analysis of the pre-branch, cell fate 1, and cell fate 2. The results indicated that the pre-branch is functionally linked to immunity, while cell fate 1 is enriched in pathways related to the extracellular matrix, adhesion, WNT, and other pathways. Cell fate 2 primarily focuses on apoptosis and matrix metalloproteinase pathways **(Figure 2I)**. We hypothesize that melanoma cells diminish the efficacy of anti-PD-1 therapy through abnormal activation of the extracellular matrix, WNT, and other pathways.

Subsequently, we performed differential expression and functional enrichment analyses within the MM_Immune subgroup between the groups **(Figure S5A-D)**. The NPD-pre group showed more robust pathways associated with lymphocyte, complement, and cell migration compared to the PD-pre group. These pathways promote the recruitment and activation of immune effector cells, enhancing the ability to kill tumors. Additionally, the NPD-post group exhibited a greater inclination towards antigen presentation, oxidative stress, and cell death compared to the NPD-pre group, indicating the strong tumor-killing ability of anti-PD-1 therapy.

To confirm the role of the MM_Immune subgroup in anti-PD-1 treatment, we utilized the Cancer Genome Atlas (TCGA)-Skin Cutaneous Melanoma (SKCM) dataset, which includes a large cohort of 471 melanoma patients. The patients were categorized into high and low groups based on their scores for the percentage of the MM_Immune subgroup. The Cancer Immunome Atlas (TCIA) provided a predictive score for ICIs treatment efficacy for each patient in the TCGA-SKCM dataset. Comparing the TCIA scores between the high and low groups, we observed no difference in the absence of anti-PD-1 and anti-CTLA-4 treatment. However, upon administration of ICIs (anti-PD-1, anti-CTLA-4, and their combination), the TCIA scores were significantly elevated (p < 0.0001) in the high-score group compared to the low-score group **(Figure 2J)**. These results suggest that the percentage of MM_Immune cells may serve as an advanced biomarker for predicting immune efficacy in patients.

### Loss of function of CD8^+^ T cells in the TME

**Figure 3:**
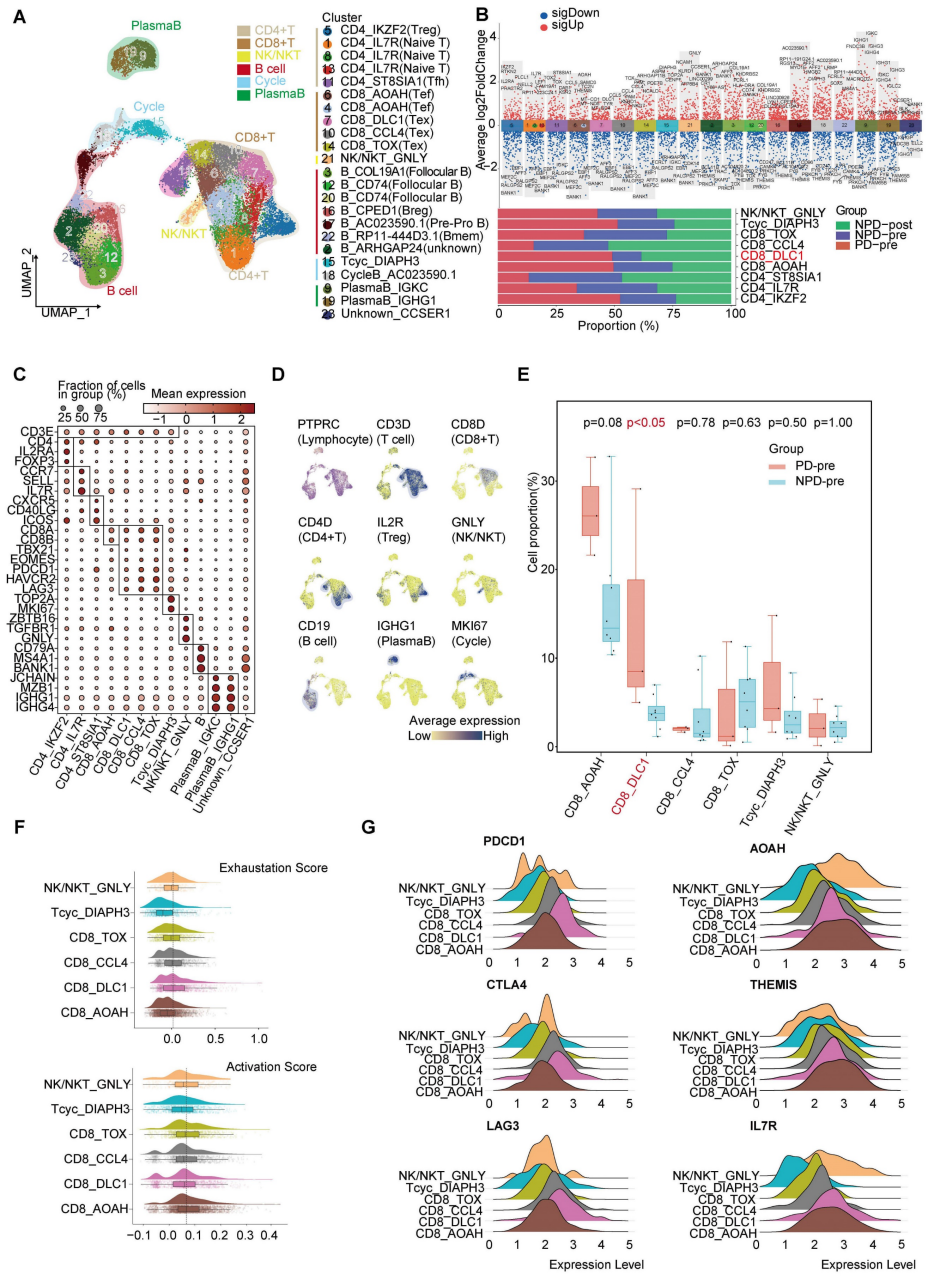
** Heterogeneity of T lymphocyte and loss of function of CD8+ T cells in the TME. A.** UMAP plot of 27 766 lymphocytes, color-coded by their associated cluster. **B.** Volcano plot (upper panel) showing the differentially expressed genes in these annotated clusters; Bar plot (lower panel) showing the proportions of T cell (including NK/NKT) subgroups. **C.** Dot plot of subgroup-specific genes of 13 lymphocyte cell types. **D.** Feature plots showing classical marker genes for the annotated 13 lymphocyte cell types. **E.** Box plot showing the proportion of annotated CD8+T and NK/NKT subgroups between PD-pre and NPD-pre groups (non-paired t test). **F.** Exhaustion Score and activation Score of CD8+T and NK/NKT subgroups. **G.** Ridge plots showing the expression levels of exhaustion and activation genes in CD8+T and NK/NKT subgroups.

Lymphocytes are integral components of the TME and play a critical role in anti-tumor immunity. To gain a deeper understanding of lymphocyte subgroups in melanoma, we employed an unsupervised clustering approach to analyze 27,766 lymphocytes, resulting in the identification of 23 distinct clusters **(Figure 3A & Figure S6A)**. We integrated subgroups with similar gene expression profiles into 19 independent subgroups with unique gene expression patterns and functions **(Figure 3B)**. T cells were annotated using previously reported cell subgroup markers based on the average expression of marker genes in the T cell subgroups **(Figure 3C-D)** 34-36. The function of T cells was also verified using GO analysis **(Figure S6B)**.

The CD8_DLC1 subgroup, characterized by a high level of DLC1 Rho GTPase activating protein (DLC1), was significantly elevated (p < 0.05) in the PD-pre group compared to the NPD-pre group **(Figure 3E)**. The distribution of other subgroups can be found in **Figure S6C**. We further observed that CD8_DLC1 cells exhibited the highest exhaustion score and a high toxic activity score, while the CD8_AOAH subgroup exhibited the lowest exhaustion score and highest toxic activity score among the four distinct CD8+ T subgroups and NK/NKT cells **(Figure 3F)**. CD8_DLC1 cells also showed high expression levels of *PDCD1*, *CTLA4*, and *LAG3*, whereas CD8_AOAH exhibited high expression levels of acyloxyacyl hydrolase (*AOAH*), thymocyte-expressed molecule involved in selection (*THEMIS*), and *IL7R* **(Figure 3G)**. These genes are known to be positively associated with melanoma prognosis, TCR and cytokine signaling in CD8+ T cells, and T cell differentiation and maintenance 37-39. We also observed variations in the activity of transcription factors across the three Tex and one Tef subgroups **(Figure S6D)**. Basic leucine zipper ATF-like transcription factor (BATF) was highly active in CD8_TOX cells and slightly active in CD8_CCL4 cells 40, 41. MAF BZIP transcription factor (MAF) exhibited high activity in the CD8_DLC1 and CD8_TOX subgroups 42. Signal transducer and activator of transcription (STAT) 4 was activated in CD8_AOAH cells 43. These findings suggest that differences in transcription factor activity may contribute to different states of CD8+ T cells, and that the regulatory activity of transcription factors within Tex is heterogeneous.

The cytotoxic capacity of CD8+ T cells in the PD-pre group was significantly lower compared to the NPD-pre group **(Figure S6E)**. To understand the mechanism of CD8+ T cell dysfunction, we conducted a comprehensive analysis of 4,041 high-quality CD8+ T and Tcyc cells, from which we established a pseudotemporal ordering. We observed that Tcyc cells were located at the root of the phylogenetic tree, with high expression of cell division cycle associated 8 (CDCA8) and centromere protein P (CENPP), while CD8_AOAH and CD8_DLC1 cells were predominantly situated at the terminal branch** (Figure S7A-B)**. Additionally, granzyme A (GZMA) and THEMIS were not present at the end of the branch represented by CD8_DLC1. Furthermore, the aggregation of CD8_AOAH and CD8_DLC1 cells gradually diverged after the branching point. These observations suggest that proliferating CD8+ T cells may undergo developmental selection at a specific pseudo-timepoint, ultimately determining their functional state. We then examined differentially expressed genes (DEGs) and enriched biological functions at the branching sites, and identified the regulation of hydrogen peroxide-induced cell death in cell fate 1 of CD8_DLC1 cells. Conversely, CD8_AOAH cells demonstrated high immune activity and resistance to apoptosis, with a notable association with B cell activation.

### CD74^+^ follicular B cells with function of antigen presentation

**Figure 4:**
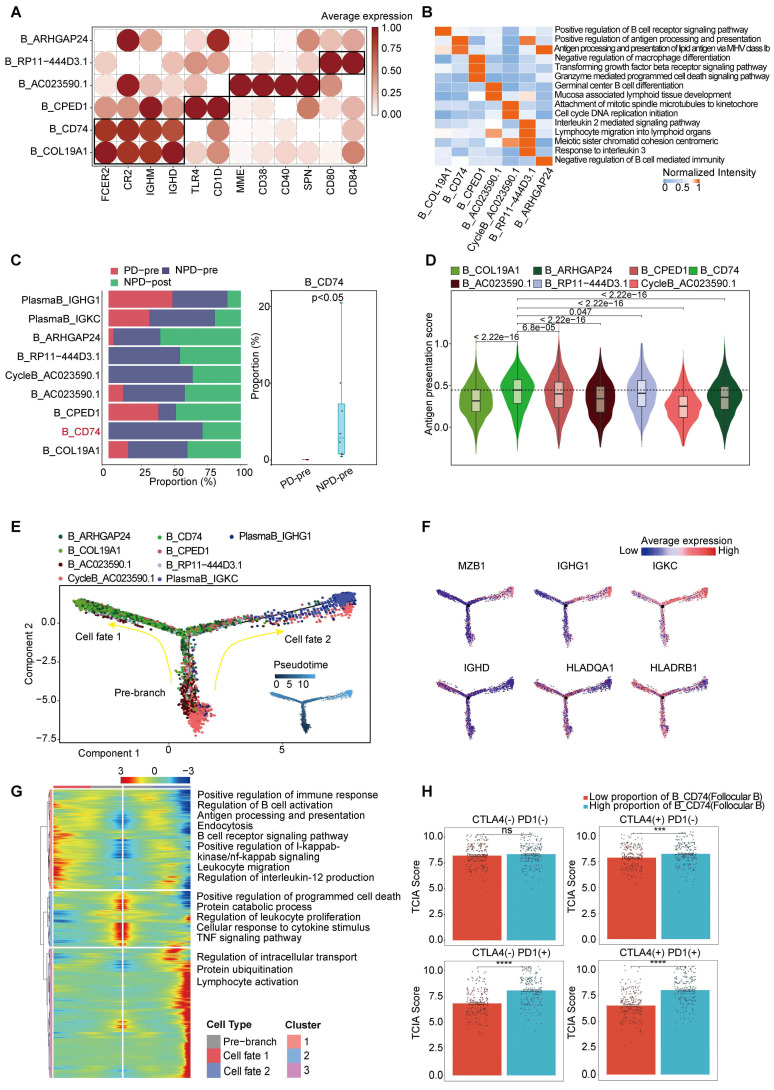
** CD74+follicular B cells with function of antigen presentation. A.** Dot plot of subgroup-specific genes of B cell types. **B.** Feature plots showing classical marker genes for the annotated B cell types. **C.** Bar plot (left panel) showing the proportions of B cell subgroups. Box plot (right panel) showing the proportion of B_CD74 (follicular B) between PD-pre and NPD-pre groups (non-paired t test). **D.** Violin plots of the antigen presentation score of each B cell subgroup (non-paired t test). **E.** Pseudo-temporal trajectory of B cells identified two distinct cell fates colored by subgroup. **F.** Pseudo-temporal trajectory of B cells colored by the expression of subgroup-specific genes. **G.** Heatmap showing the enriched pathways in cellfate1 and pre-branch. **H.** Bar plots of TCIA score of TCGA patients categorized into high and low groups based on their score of the percentage of the B_CD74 subgroup (non-paired t test).

Based on the existing literature, we identified and annotated six distinct subgroups of B cells **(Figure 4A)** 44, 45. Among these subgroups, B_CD74 (follicular B) stood out as it exhibited a significant correlation with antigen presentation, as indicated by the increased expression of CD74 and major histocompatibility complex (MHC) class II molecules, such as HLA-DRA **(Figure 3B & Figure 4B)**. Previous studies have rarely observed B cells mediating antigen presentation through MHCII molecules. However, recent research suggests that enhancing the MHCII-mediated antigen presentation pathway in B cells can promote tumor-specific T cell responses and inhibit tumor progression 46. Notably, the B_CD74 subgroup was absent in the PD-pre group, while its proportion in the NPD-pre group showed significant variation (p < 0.05), ranging up to approximately 20% **(Figure 4C)**. We calculated antigen presentation scores and found that this subgroup had the highest scores **(Figure 4D)**. Subsequently, we conducted pseudo-time analysis of 10,763 highly qualified B cells. The B_CD74 subgroup was situated at the terminal branch, with MHCII molecules, such as HLA-DQA1 and HLA-DRB1, distributed along the trajectory of cell fate 1, indicating a potent and precise function **(Figure 4E-F)**. Further analysis of the branches in the trajectories revealed that B cells in the pre-branch were enriched in the regulation of leukocyte proliferation and the TNF signaling pathway. As cells progressed from the pre-branch to cell fate 1, some of these cells differentiated into B_CD74 cells, gradually activating the B cell receptor and acquiring leukocyte migration, endocytosis, and antigen presentation capabilities **(Figure 4G)**. Plasma cells were enriched at the end of cell fate 2, indicating a higher degree of differentiation consistent with their specialized biological function **(Figure 4E)**. The main features of this branch included intracellular transport, protein ubiquitination, and lymphocyte activation, suggesting that plasma cells were in an activated state and actively regulated intracellular protein metabolism and transport **(Figure 4G)**.

CycleB_AC023590.1 and B_RP11-444D3.1 were also found to be missing in the PD-pre group, which may indicate local scarcity of B cells in the PD-pre group from the perspective of local B cell generation **(Figure 3B)**. As shown in **Figure 4E**, CycleB_AC023590.1 was primarily located at the root of the developmental tree, exhibiting a lower degree of differentiation and primarily responsible for proliferation. It may represent the main source of in situ B cell generation. B_RP11-444D3.1 was more evenly distributed throughout the root, middle, and end of the developmental tree, but in smaller numbers. This suggests that this subpopulation has diverse functions and varying degrees of differentiation, rather than representing a specific function. In contrast, B_CD74 is characterized by its clear antigen presentation function. Therefore, B_CD74 was selected as the core subpopulation for characterizing B cell function.

Subsequently, we evaluated the relationship between B_CD74 cells and anti-PD-1/CTLA-4 treatment. Patients were divided into high and low groups based on the percentage of B_CD74 cells, with the high group exhibiting higher TCIA scores **(Figure 4H)**. These results indicate that B_CD74 cells play important roles in anti-tumor immunity and are closely associated with anti-PD-1 therapy.

### The strongest immune activation subgroup in myeloid cells

Expansion of myeloid cells in the TME, including dendritic cells (DCs), macrophages, monocytes, and mast cells, is a dynamic process that can either promote or suppress immune responses **(Figure S8A)** 47. We employed unsupervised clustering to categorize myeloid cells into nine distinct groups, each characterized by specific markers **(Figure S8B-C)**. Among these groups, migratory DCs (migDCs) exhibited notable expression of CD200 molecule (*CD200*) and suppressor of cytokine signaling 2 (*SOCS2*); type 1 conventional dendritic cells (cDC1s) expressed C-type lectin domain containing (*CLEC*) 9A and *BATF3*; cDC2s expressed *CLEC10A* and CD1c molecule (*CD1C*); pDCs expressed *CLEC4C* and *IL3RA*; and monocytes expressed CD14 molecule (*CD14*). In contrast to monocytes, macrophages exhibited increased expression of complement C1q A chain (*C1QA*) and complement C1q B chain (*C1QB*), whereas mast cells exhibit high levels of KIT proto-oncogene, receptor tyrosine kinase (*KIT*) and PBX homeobox 1 (*PBX1*). Proliferating cells were characterized by *TOP2A* and *CDCA8* expression, and a subgroup lacking classification markers was identified and labeled as Unknown 48-50. Additional marker genes are shown in **Figure S8D**. Overall, the proportions of mast cells, macrophages, and monocytes were similar between the PD-pre and NPD-pre groups. However, the NPD-pre group showed enrichment of DCs compared to the PD-pre group, prompting further investigation into the potential contribution of DCs to anti-PD-1 efficacy** (Figure S8E)**. We examined the distribution of functional genes, including MHC molecules, immune checkpoints, and chemokines, within the DC subgroups **(Figure S8F)**. The migDCs exhibited proclivity towards expressing MHC I molecules and immune checkpoints, such as *PDCD1* and matrix metallopeptidase (*MMP*). Conversely, cDC1s expressed both MHC I and II molecules, as well as excitatory and immune checkpoints, such as *CTLA4*. Additionally, the pDCs demonstrated expression of interferon (IFN)-γ. Compared with other DCs, cDC2s expressed MHC II molecules, which are the most abundant chemokines. Meanwhile, cDC2s express low levels of immune checkpoints, such as *PDCD1* and *CTLA4*, but highly express *TNF* and immune-active factors, such as *ICOS* and TNF superfamily member 18 (*TNFSF18*).

Based on the aforementioned analysis, we concluded that the cDC2_RTN1 subgroup possessed the most robust immune activation capacity among the DC subgroups. Interestingly, the proportion of cDC2_RTN1 cells was significantly higher (p < 0.05) in the NPD-pre group compared to the PD-pre group **(Figure S8G)**. Furthermore, we observed a more intense expression of immune genes in the NPD-pre group compared to the PD-pre group **(Figure S8H)**, suggesting that cDC2s in the NPD-pre group had a more potent immune activation ability. The activation of KLF transcription factor 6 (KLF6), a pivotal regulator of favorable immune responses in dendritic cells associated with antigen presentation, might contribute to the remarkable immune activation capacity of cDC2s **(Figure S8I)**. Thus, the cDC2 subgroup may serve as a primary determinant of a favorable prognosis. To validate this, patients in the TCGA-SKCM dataset were divided into high and low groups based on the percentage of cDC2_RTN1 cells. Those in the high group exhibited significantly higher (p < 0.001) TCIA scores for anti-PD-1 therapy, indicating the important role of cDC2_RTN1 cells in the effectiveness of anti-PD-1 treatment **(Figure S8J)**. Therefore, we determined that the cDC2_RTN1 subgroup was the strongest immune activator among the DC subgroups and could serve as a reliable biomarker for predicting the prognosis of immune checkpoint inhibitor (ICI) treatment.

### An iCAF subgroup characterized by immune chemotaxis

**Figure 5:**
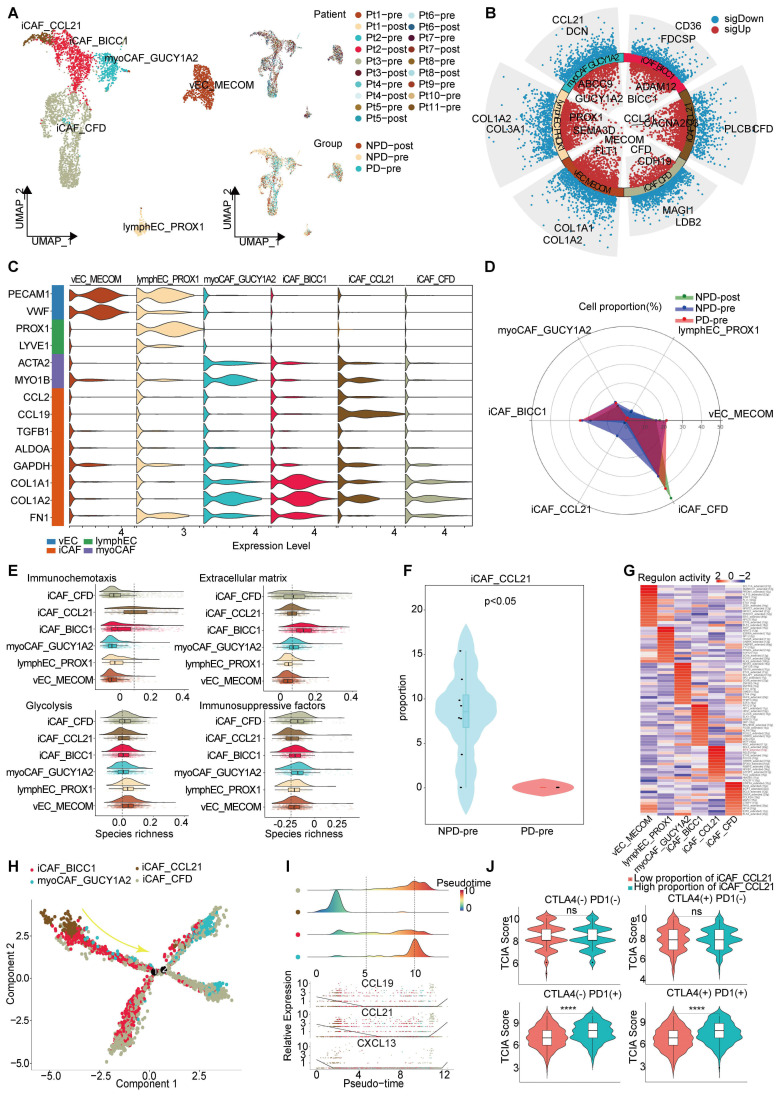
** Immune driving and suppressing capabilities of iCAF_CCL21. A.** UMAP plot (left panel) of 4 369 interstitial cells, color-coded by their associated cluster; UMAP plot (upper right panel) interstitial cells, color-coded by patient origin; UMAP plot (lower right panel) interstitial cells, color-coded by groups. **B.** Volcano plot showing the differentially expressed genes in the six annotated clusters. **C.** Violin plots showing the subgroup-specific genes of each subgroup. **D.** Radar chart showing the proportion of annotated six subgroups between groups. **E.** Immunochemotaxis, extracellular matrix, glycolysis, immunosuppressive score of interstitial cell subgroups.** F.** Violin plot showing the proportion of iCAF_CCL21 between PD-pre and NPD-pre groups (non-paired t test). **G.** Dot plot of the t-values of AUC scores of expression regulation by transcription factors, as estimated using SCENIC, per subgroup of interstitial cells. **H.** Pseudo-temporal trajectory of CAFs colored by subgroup. **I.** The cell density distribution (upper panel) of the pseudotime-ordered CAFs; The expression of chemokine (lower panel) of the pseudotime-ordered CAFs. **J.** Violin plots of TCIA score of TCGA patients categorized into high and low groups based on their score of the percentage of the iCAF_CCL21 subgroup (non-paired t test).

Through unsupervised clustering and classical markers, we identified six subgroups of interstitial cells **(Figure 5A)**: vascular endothelial cells with elevated Von Willebrand factor (VWF) expression, lympho-endothelial cells with heightened prospero homeobox 1 (PROX1) expression, myofibroblastic cancer-associated fibroblast (myoCAFs) primarily expressing actin alpha 2 (ACTA2) and myosin IB (MYO1B), and iCAF characterized by chemokines and immunosuppressive factors, each exhibiting distinct gene expression patterns** (Figure 5B-C)**. Subsequently, we found that the NPD-pre group exhibited a greater number of iCAFs with elevated levels of CCL21 (maintaining the stability and proper localization of immune cell populations is of utmost importance for the immune system 51) expression (named iCAF_CCL21), prompting us to hypothesize that this particular subgroup may confer a favorable impact on anti-tumor immunity **(Figure 5D)**. Previous studies have shown that cancer-associated fibroblasts (CAFs) can have both promoting and inhibiting effects on tumor drug resistance. The immune regulation contradiction can be summarized by four primary pathways: immune chemotaxis and activation of anti-tumor immunity, extracellular matrix secretion to impede immune infiltration, glucose metabolism acidification, and TME inhibition of immune activity and immunosuppressive factor secretion 52, 53. To assess the immunomodulatory potential of iCAF_CCL21, we assigned scores to all stromal cells based on these four pathways. iCAF_CCL21 cells exhibited the highest immune chemotaxis score among all CAF subgroups, while displaying the lowest scores for extracellular matrix, glucose metabolism, and immunosuppressive factors **(Figure 5E)**. Notably, iCAF_CCL21 was absent in the PD-pre group, suggesting that the immune-related pathway mediated by this subgroup was suppressed in the PD-pre group, including its intrinsic and intercellular communication-mediated immune effects **(Figure 5F)**. Based on the gene expression characteristics of the iCAF_CCL21 subgroup and its enrichment in the NPD-pre group, we believe that this subgroup is also a key factor driving immune activation. Through SCIENIC analysis, we identified high transcriptional activity of the transcription factor IRF8 in iCAF_CCL21, which plays a positive role in the induction and activation of CD8+ T cells and DCs** (Figure 5G)** 54.

Pseudo-time trajectory analysis revealed a gradual decline of iCAF_CCL21 cells along the pseudo-time trajectory **(Figure 5H)**. Chemokines such as CCL19, CCL21, and CXCL13 in the CAF cell population disappeared as the proportion of this subgroup decreased, suggesting a reduction in the capacity to attract T, B, and DC cells within the TME and potentially inhibiting the formation of tertiary lymphoid structures **(Figure 5I)** 55. Previous research has shown that patients lacking tertiary lymphoid structures tend to have poor response to immune checkpoint inhibitor (ICI) treatment 56. However, the markers of iCAF_CCL21 did not predict the efficacy of anti-CTLA-4 immunotherapy, but showed significant differentiation between TCGA-SKCM patients with low and high proportions of both anti-PD-1 and anti-PD-1/anti-CTLA-4 combination therapies **(Figure 5J)**. These results suggest that the absence of iCAF_CCL21 is closely related to poor response to anti-PD-1 treatment in the PD-pre group.

### Differences in cell numbers of subgroups between groups by immunofluorescence

**Figure 6:**
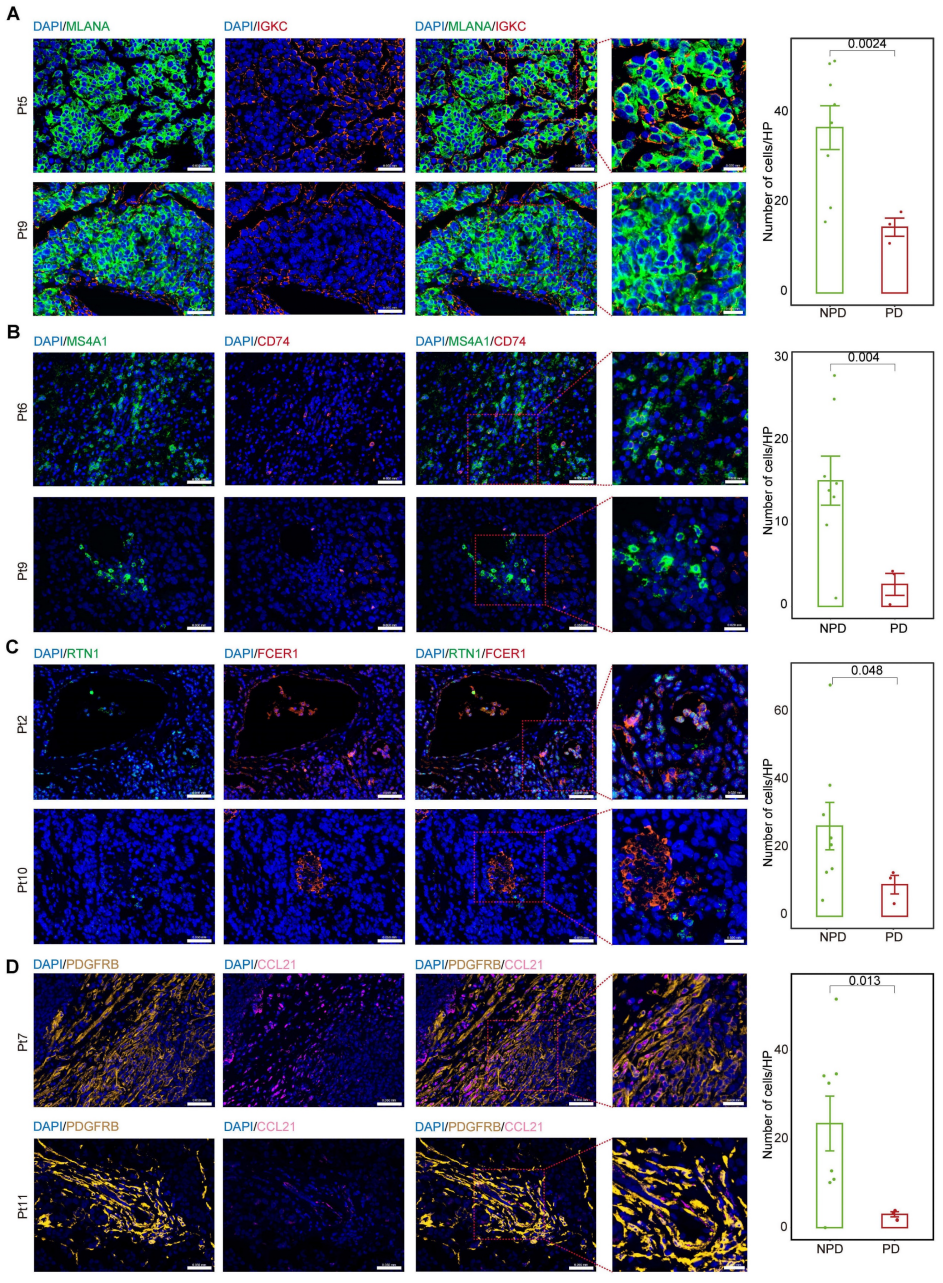
** MM_Immune, B_CD74, cDC2_RTN1 and iCAF_CCL21 maintain the molecular features between the PD-pre and NPD-pre groups. A.** Representative images and quantification of immunostaining with anti-MLANA and anti-IGKC antibodies on PD-pre tumor (n = 3) and NPD-pre tumor (n = 8); scale bar: 50 μm (left); 20 μm (right) (non-paired t test); High power field (HP): a square with a side length of 140 μm, selected under high power field(80x), has a field of view area of 0.0196 mm².** B.** Representative images and quantification of immunostaining with anti-MS4A1 and anti-CD74 antibodies on PD-pre tumor (n = 3) and NPD-pre tumor (n = 8); scale bar: 50 μm (left); 20 μm (right) (non-paired t test). High power field (HP): a square with a side length of 140 μm, selected under high power field(80x), has a field of view area of 0.0196 mm². **C.** Representative images and quantification of immunostaining with anti-RTN1 and anti-FCER1 antibodies on PD-pre tumor (n = 3) and NPD-pre tumor (n = 8); scale bar: 50 μm (left); 20 μm (right) (non-paired t test). High power field (HP): a square with a side length of 140 μm, selected under high power field(80x), has a field of view area of 0.0196 mm². **D.** Representative images and quantification of immunostaining with anti-PDGFRB and anti-CCL21 antibodies on PD-pre tumor (n = 3) and NPD-pre tumor (n = 8); scale bar: 50 μm (left); 20 μm (right) (non-paired t test). High power field (HP): a square with a side length of 140 μm, selected under high power field(80x), has a field of view area of 0.0196 mm².

We calculated the markers for the MM_Immune, B_CD74, cDC2_RTN1, and iCAF-CCL21 subgroups (see Methods). Immunofluorescence staining was performed for the MM_Immune marker, IGKC, and the melanoma marker, Melan-A (MLANA), in formalin-fixed paraffin-embedded tissues of melanoma samples** (Figure 6A & Figure S9A)** demonstrated an increased MM_Immune cell ratio in the NPD-pre group. Membrane spanning 4-domains A1 (MS4A1)+/CD74+ **(Figure 6B & Figure S9B)**, RTN1 +/Fc epsilon receptor I (FCER1)+**(Figure 6C & Figure S9C)**, and platelet derived growth factor receptor beta (PDGFRB) +/CCL21+ cells **(Figure 6D & Figure S9D)** were stained in the same manner. These results confirmed that MM_Immune, B_CD74, cDC2_RTN1, and iCAF-CCL21 cells were more enriched in the NPD-pre group compared to the PD-pre group, consistent with the results of the aforementioned analysis.

### Anti-tumor immunity maintained by iCAF_CCL21

**Figure 7:**
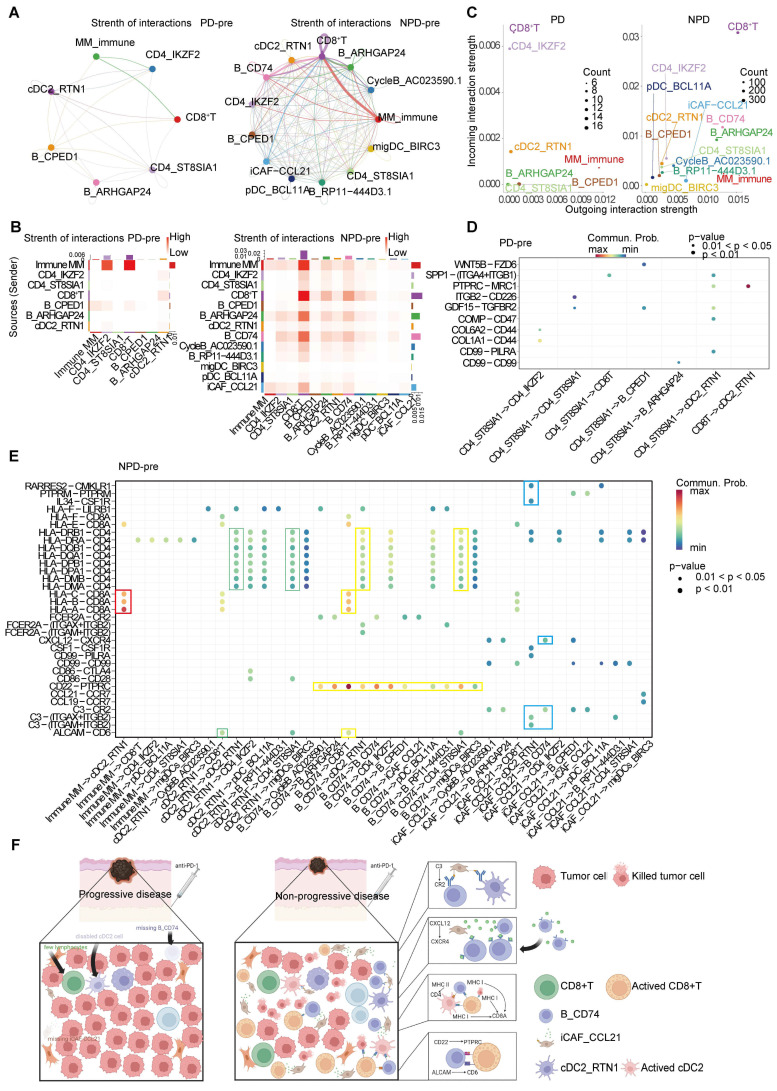
** The inter-cellular communication networks in the tumor ecosystem of melanoma. A.** The interaction strength of selected 13 cell types in PD-pre group (left panel) compared with NPD-pre group (right panel). **B.** Heatmaps of overall outgoing and incoming signaling patterns in PD-pre (left panel) and NPD-pre groups (right panel). **C.** Scatter plots showing the incoming and outcoming interaction strength in the ecosystem of PD-pre (left panel) and NPD-pre groups (right panel). **D.** Dot plot showing significantly upregulated interaction pathways in PD-pre group. **E.** Dot plot showing significantly upregulated interaction pathways in NPD-pre group **F.** Schematic diagram illustrating the differences in intercellular communication between the PD-pre and NPD-pre groups.

To detect crosstalk between different cell types in the TME, we investigated the cell-cell interaction network. We focused on cell types that showed significant differences between the PD-pre and NPD-pre groups, as well as CD8+ T cells. Comparing the PD-pre group to the NPD-pre group, we observed more communication and immune-related signals in the NPD-pre group **(Figure 7A-B)**.

Specifically, MM_Immune cells were the primary signal senders, while CD8^+^ T cells and Tregs were the main signal receivers in the PD-pre group. In the NPD-pre group, MM_Immune, B_CD74, CD8+ T, and iCAF_CCL21 cells were identified as the primary signal senders, while CD8+ T and B_CD74 cells were the main signal receivers **(Figure 7C)**. Interestingly, a large number of immune-related interactions were detected in the NPD-pre group, indicating an increase in immune-related signaling pathways **(Figure 7D-E)**. We found that MM_Immune cells mainly interacted with CD8+ T cells through MHC I molecules. Additionally, B_CD74 and cDC2s played immunoregulatory roles. B_CD74 cells communicated with multiple cell types through the protein tyrosine phosphatase receptor type C (PTPRC)-CD22 molecule (CD22) pathway, potentially facilitating the maturation and activation of immune cells **(Figure S10A)**. MHC I and MHC II molecules induced antigen presentation and increased the activity of CD8+ T, cDC2_RTN1, and other CD4+ T cells **(Figure S10B-C)**. Furthermore, the binding of CD6 molecule (CD6) and activated leukocyte cell adhesion molecule (ALCAM) to the surface of CD8+ T cells promoted their activation and infiltration** (Figure S10D)**. Notably, B_CD74 cells were recruited and activated by iCAF_CCL21 cells through the complement pathway **(Figure S10E)**. cDC2_RTN1 was activated via the complement pathway as well **(Figure S10F)**. A summarized diagram illustrating the differences in intercellular communication between the PD-pre and NPD-pre groups is presented in **Figure 7F**. Previous studies have found that fibroblast-like cells may recruit B cells through the CXCL12-CXCR4 axis 57. In the immune microenvironment of metastatic colorectal cancer, impaired antigen presentation by B cells through MHC II molecules has also been observed 58. Furthermore, CD6 activation of T cells promotes TCR diversity 59, while CR2-mediated complement pathway activation enhances B cell-driven activation of tumor-specific CD8+ T cells in anti-tumor immunity 60. Our data suggests that these previous findings may be interconnected through specific crosstalk pathways. When the recruiter CAF_CCL21 is deficient, the loss of the CXCL12-CXCR4 axis recruitment impairs the infiltration of B_CD74 responders, disrupting B cell-mediated antigen presentation and suppressing the anti-tumor effect of CD8+ T cells. In this process, the absence of CAF_CCL21 also impairs complement-dependent CR2 activation of B_CD74, while the dysfunction of B_CD74 weakens CD6-dependent activation of CD8+ T cells, thereby attenuating their involvement in anti-tumor immunity. This intricate network becomes one of the key factors determining the prognosis of anti-PD-1 therapy in malignant melanoma.

### Predictive potential of Comprehensive.sig

**Figure 8:**
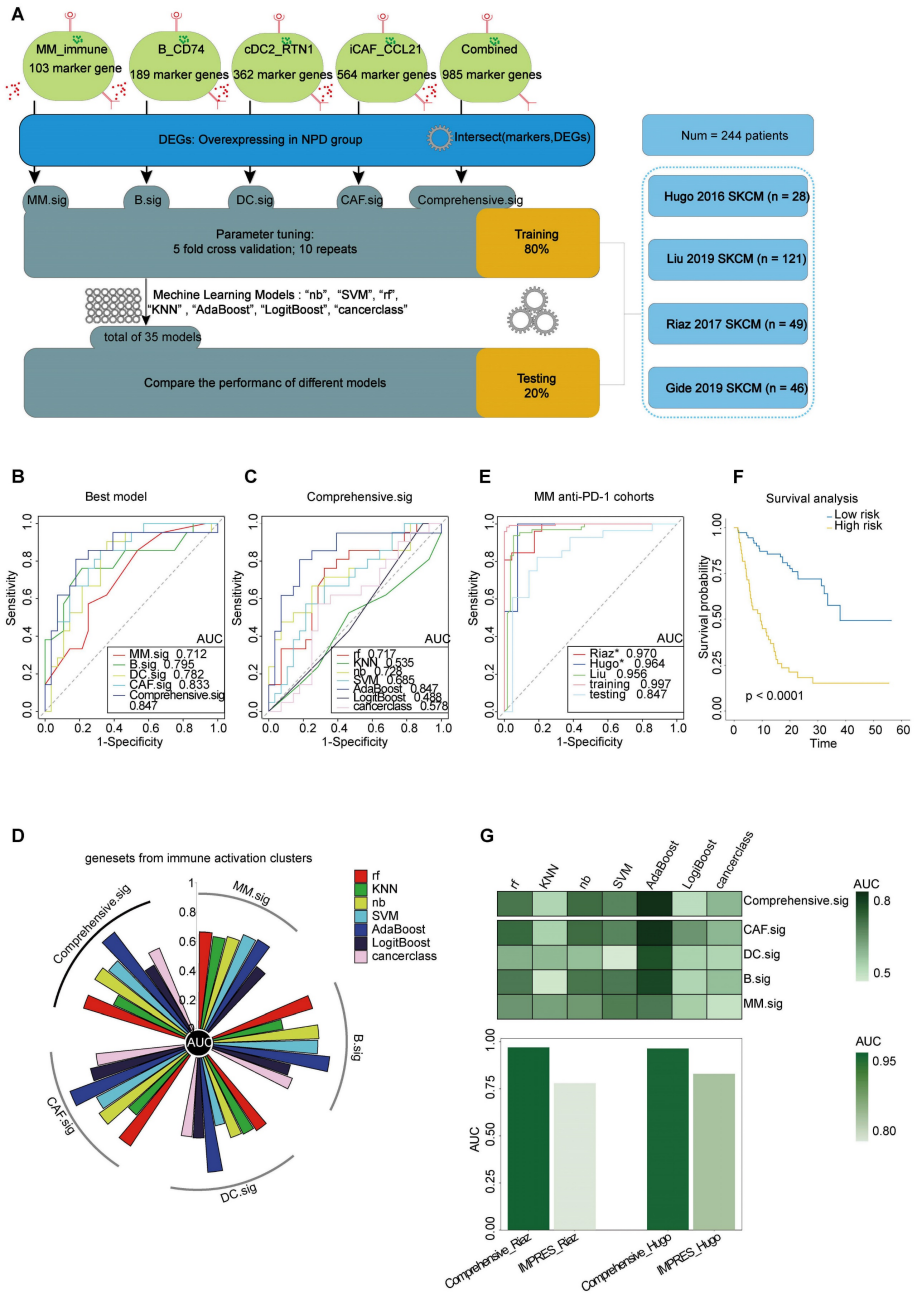
** Prediction of ICIs treatment outcomes using Comprehensive.sig. A.** Flow chart of training and testing the Comprehensive.sig model constructed using machine learning process. In the training set, we applied 10-time repeated 5-fold cross-validation for parameters tuning of different machine learning algorithms. In the testing set, AdaBoost algorithm with best AUC was kept as the fnal Comprehensive.sig model. **B.** Comparison of multiple ROC plot depicting the performance of each best model of MM.sig, B.sig, DC.sig, CAF.sig and Comprehensive.sig in the testing set. **C.** Comparison of multiple ROC plot depicting the performance of different machine learning algorithms of Comprehensive.sig in the testing set. **D.** Circos plot (upper panel) depicting the performance of models of MM.sig, B.sig, DC.sig, CAF.sig and Comprehensive.sig based on different machine learning algorithms in the testing set; Heatmap (lower panel) depicting the performance of models of MM.sig, B.sig, DC.sig, CAF.sig and Comprehensive.sig based on different machine learning algorithms in the testing set. **E.** Comparison of multiple ROC plot depicting the performance of Comprehensive.sig in multiple MM anti-PD-1 cohorts. **F.** Kaplan-Meier curves comparing OS between High-risk and Low-risk patients in validation and testing set. “PD” and “NPD” predicted by the Comprehensive.sig Model was defined as “High-risk” and “Low-risk” patients respectively. **G.** Bar plot depicting the AUC values of Comprehensive.sig and IMPRES.Sig in the same SKCM cohort (Raiz 2017 and Hugo 2016).

To improve the prediction of anti-PD-1 efficacy in melanoma, we utilized bulk RNA-Seq data from published studies 61-64 on anti-PD-1 ICI cohorts. Pre-treatment samples from these cohorts were carefully selected and analyzed. The cohorts were divided into two datasets: a training set (n = 195) and a testing set (n = 49). The analytical process is outlined as follows **(Figure 8A)**: First, we calculated the markers for the four subgroups (see Methods) to generate four gene lists: G_MM, G_B, G_DC, and G_CAF. Next, we performed a t-test to identify differentially expressed genes (DEGs) enriched in non-progressive disease (NPD) patients from the ICI cohort (p < 0.05), resulting in a gene list, Gx. We then intersected Gx with G_MM, G_B, G_DC, and G_CAF to obtain four gene sets that represented the upregulated immune activation subgroups in the NPD-pre group. These gene sets were named MM.sig, B.sig, DC.sig, and CAF.sig. Additionally, to investigate whether a combination of these gene sets could encompass a wider range of biological factors, we merged them to create a fifth gene set called "Comprehensive.sig". Subsequently, we trained the model using seven different machine learning algorithms for each gene set, and performed 10-time-repeated 5-fold cross-validation to optimize the parameters in each model. This yielded a total of 35 models. The performance of these models was evaluated and compared using the area under the curve (AUC) in a test cohort. The best model, which used the combined gene set, achieved the highest AUC of 0.847 **(Figure 8B)** and was selected as the final model, referred to as the Comprehensive.sig model **(Figure 8C-D)**. Furthermore, we evaluated the AUC of the model in each cohort and training set, and the results demonstrated that all AUC values exceeded 0.95 **(Figure 8E)**. To evaluate the model's predictive ability for overall survival, we divided patients who received ICI treatment into low-risk and high-risk subgroups based on the predicted values of "NPD" and "PD", respectively. The Kaplan-Meier analysis of overall survival **(Figure 8F)** revealed that the low-risk subgroup had significantly longer overall survival (p < 0.0001). The predicted results were consistent with the inference from xCell **(Figure S11A)**. Zhang et al. compared melanoma-specific models and found that IMPRES.Sig was the best model 65, 66. When comparing our model with the AUC of IMPRES.Sig in the same cohort, our model exhibited a higher AUC in terms of predicting anti-PD-1 response **(Figure 8G)**.

## Discussion

We conducted a comprehensive analysis of snRNA-seq data from an anti-PD-1 treatment cohort to gain insights into the TME in patients with varying treatment outcomes. Notably, we observed an increase in lymphocytes and a decrease in myeloid cells in the TME of patients in the non-progressive disease (NPD)-pre group compared to the progressive disease (PD)-pre group. We analyzed various cell types, including tumor cells, lymphocytes, myeloid cells, and interstitial cells, and identified four subgroups that were significantly enriched in the NPD-pre group and exhibited strong immune-driven characteristics. To validate the potential of these subgroups as biomarkers for predicting the prognosis of anti-PD-1 treatment, we verified our findings using TCGA-SKCM and TCIA data. Immunofluorescence staining confirmed that these cells were more abundant in the tissues of patients in the NPD-pre group. Furthermore, through cell-cell interaction analysis, we observed that the absence of iCAF_CCL21 cells in the PD-pre group may inhibit chemotaxis and weaken immune cells. This could explain the scarcity of B_CD74 cells and the absence of cDC2_RTN1 cells in the PD-pre group. Ultimately, we determined that combining the four subgroups into a gene set resulted in the most accurate machine learning model for predicting the response to anti-PD-1 treatment.

Over the past decade, anti-PD-1 treatment has shown superior efficacy compared to high-dose interferons, marking a new era in immune checkpoint inhibitors (ICIs) treatment 67. However, the overall response rate (ORR) for melanoma remains below 50%, leading to increased interest in predicting treatment outcomes prior to medication administration. Various biomarkers have been identified to predict the response to anti-PD-1 treatment 68-70. Although traditional whole-exome sequencing and bulk RNA sequencing can identify a significant proportion of biomarkers, such as tumor mutational burden and DEGs, their accuracy is limited due to the inability to accurately assess gene expression from multiple distinct cells. This limitation results in the inclusion of irrelevant features when building the model, leading to an AUC usually below 0.75. Single-cell sequencing provides a precise gene distribution map of a sample, allowing for the identification of additional biomarkers. When combined with bulk RNA data, this approach narrows down the range of features and increases the AUC to 0.8 or higher.

We identified four main functions in tumor cells related to antigen presentation, EMT, proliferation, and stress. We suggested that antigen presentation characteristics are closely linked to prognosis, with this signature diminishing as tumors progress. Importantly, the disparity between the PD-pre and NPD-pre groups can be attributed to the upregulation of antigen prognostic characteristics and stronger lymphocyte-related functions in the NPD-pre group. These findings are consistent with those of Lina et al., who emphasized the critical role of antigen presentation in activating autologous CD8^+^ T cells in the prognosis of ICIs treatment 71. According to Lavinia et al., the loss of heterozygosity resulting from frequent genomic alterations in advanced tumors frequently affects antigen presentation 72. Therefore, we propose that the antigen presentation signature in melanoma may serve as a target to enhance the response to ICIs treatment. Additionally, when activated by the extracellular matrix and the Wnt pathway, melanoma cells are associated with a poorer prognosis, which aligns with previous studies demonstrating the induction of immune tolerance by these factors in melanoma.

Despite the enrichment of melanoma cells with antigen-presenting function in the NPD group, enhancing the immunogenicity of these tumors alone is insufficient to explain the observed stronger anti-tumor immunity in the NPD-pre group. CD8^+^ T cell exhaustion in tumor immunity leads to immunosuppression, which is associated with an unfavorable patient prognosis 73. We observed heterogeneity in the distribution of transcription factors within exhausted T cells undergoing oxidative stress-induced cell death. The antigen-presenting function of B cells in the cellular immune process is crucial, despite their classification as nonclassical antigen-presenting cells 74. We found that the B_CD74 subgroup, which possesses antigen-presenting ability, accounted for up to 20% (maximum proportion) of lymphocytes in the NPD-pre group, but was completely absent in the PD-pre group. We also confirmed cDC2_RTN1 as the subgroup with the highest immune driving potential in dendritic cells (DCs), found it to be disabled in the PD-pre group, and identified benign subgroups in cancer-associated fibroblasts (CAF) with the strongest immune chemotactic ability and the lowest immunosuppressive potential. We discovered a deficiency of highly expressed chemokine factor CAF_CCL21 in the tumor microenvironment of PD patients. The disruption of the CXCL12-CXCR4 axis and the attenuation of complement pathway activation may be the fundamental reasons for the absence of B_CD74 and the disability of cDC2_RTN1, further influencing the activation of CD8+ T cells through the ALCAM-CD6 axis and antigen presentation. In fact, we observed higher cytotoxicity and activation scores in CD8+ T cells from the NPD group compared to the PD group, providing us with a framework to explore the intricate anti-tumor network within the human body and integrate previous discoveries. In conclusion, this framework provides a foundation for developing an ICI treatment prognostic model that incorporates various subgroups.

Our objective was to develop a customized model for predicting the response to anti-PD-1 drugs. With the proliferation of machine-learning algorithms, researchers now have a wide range of options to choose from. However, relying on a single approach may not yield optimal results. Drawing from the work of Zhang et al. 65, we recognized that random forest, K-Nearest Neighbor (KNN), Naïve Bayes, and other models have their own strengths and weaknesses across different datasets. Therefore, we selected seven commonly used models for our analysis. In contrast to previous studies, our analysis led to the identification of four distinct subgroups. We then compared the accuracy of models constructed from each subgroup and ultimately chose the best model that integrated all four subgroups. As a result, our model takes into account the biological factors of all four subgroups, rather than just one. In terms of predicting the response to anti-PD-1 treatment, our model outperforms Xiong et al.'s ImmuneCells.Sig, which was developed based on a single subgroup (γδT) and the cancerclass package (AUC: 0.97 vs. 0.96 in Riaz 2017; 0.956 vs. 0.88 in Liu 2019; 0.997 vs. 0.98 in the training set) 12. Furthermore, our model demonstrates superior performance compared to Auslander N's IMPRES.Sig in terms of anti-PD-1 prediction (AUC: 0.97 vs. 0.78 in Riaz 2017; 0.956 vs. 0.83 in Hugo 2016) 66.

### Limitations

Our study has several limitations. Firstly, we employed a single-cell nuclear sequencing method to obtain data, which may be less accurate in identifying immune cells compared to the standard single-cell sequencing approach. This could potentially result in minor discrepancies between our immune cell analysis and the actual composition 75. Secondly, neoadjuvant therapy involving the use of anti-PD-1 monoclonal antibodies in combination with sensitizers or chemotherapy has become a common practice in China to impede the progression of melanoma. This makes it increasingly challenging to have a large cohort of patients who receive only anti-PD-1 antibodies for the treatment of melanoma in clinical practice. As a result, we were unable to obtain a sizable internal validation cohort to assess the accuracy of our model. In the future, we plan to continue following up with patients who have chosen anti-PD-1 monotherapy to expand our cohort and conduct further research. Additionally, the predictive value of our model will need to be validated through prospective clinical trials investigating the efficacy of ICIs.

## Conclusions

In this study, we conducted an analysis of snRNA-seq data from a cohort of patients treated with anti-PD-1 therapy, revealing the presence of heterogeneity in tumor cells, lymphocytes, myeloid cells, and stromal cells. Through our investigation, we identified immune-driven subgroups that have the potential to serve as biomarkers impacting the prognosis of ICIs treatment. Moreover, we developed an advanced model that integrates multiple subgroups as biological factors to predict the response to anti-PD-1 therapy. This model provides valuable insights and guidance for the selection and combination of anti-PD-1 drugs in the ICIs treatment of MM.

## Materials and Methods

### Human tumor specimens

All melanoma samples were collected from the Department of Musculoskeletal Oncology, Fudan University Shanghai Cancer Center, after obtaining informed consent from the patients. Tumor samples without obvious hemorrhage, necrosis, or electrical burn were collected during the surgery.

### Single nucleus RNA-seq preparation of single-cell suspensions

Fresh primary lesions of 11 patients were isolated immediately following tumor resection and transferred to a 50 mL centrifuge tube filled with precooled RPMI 1640 medium with 0.04 % bovine serum albumin (BSA, Gibco, Carlsbad, CA, USA). They were then quickly transported on ice to the FDZSH laboratory to minimize the ischemic time. Samples were cut into 1 mm3 pieces, followed by enzymatic digestion using the Miltenyi Tumor Dissociation Kit (Miltenyi, Bergisch Gladbach, Germany). The samples were then centrifuged at 300 g for 30 s, and the supernatant was discarded. Next, 1× PBS (calcium and magnesium free) containing 0.04 % BSA (400 µg/ml) was added and then centrifugation at 300 g for 5 min. The cell pellet was resuspended in 1 ml red blood cell lysis buffer and incubated for 10 min at 4 ℃. The samples were then resuspended in 1 ml PBS containing 0.04 % BSA. The samples were then filtered using Scienceware Flowmi 40-µm cell strainers (VWR). Finally, 10 µL of suspension was counted under an inverted microscope with a hemocytometer. Trypan blue was used to quantify the live cells.

### Single nucleus isolation and sequencing

Nuclei were isolated using the ShBio Nuclei Isolation Kit (SHBIO, #52009-10, Shanghai, China). Nuclei were counted using a cell counter (Thermo Fisher Scientific). Using a Chromium Single Cell 3′ Library and Gel Bead Kit v3 (10X Genomics), nuclei were immediately loaded onto a Chromium Single Cell Processor (10X Genomics) to barcode the RNA from single nuclei. Sequencing libraries were constructed according to the manufacturer's instructions (10X Genomics) and sequenced using a NovaSeq 6000 sequencing system (Illumina, 20012866) 76.

### Single nucleus RNA-seq data processing

Reads were processed using the Cell Ranger 3.0.1 pipeline with default and recommended parameters. FASTQs generated from Illumina sequencing output were aligned to the human genome, version GRCm38, using the STAR algorithm 77. Next, Gene-Barcode matrices were generated for each individual sample by counting UMIs and filtering non-cell associated barcodes.Finally, we generate a gene-barcode matrix containing the barcoded cells and gene expression counts. This output was then imported into the Seurat_3.0.2 R toolkit for quality control and downstream analysis of our single cell RNAseq data. All functions were run with default parameters, unless specified otherwise. We excluded cells with fewer than 200 or more than 6000 detected genes (where each gene had to have at least one UMI aligned in at least three cells). The expression of mitochondria genes was calculated using PercentageFeatureSet function of the seurat package 78. To remove low activity cells, cells with more than 10 percent expression of mitochondria genes were excluded. The normalized data (NormalizeData function in Seurat package) was performed for extracting a subset of variable genes. Variable genes were identified while controlling for the strong relationship between variability and average expression. Next, we integrated data from different samples after identifying 'anchors' between datasets using FindIntegrationAnchors and IntegrateData in the seurat package. Then we performed principal component analysis (PCA) and reduced the data to the top 30 PCA components after scaled the data. We visualized the clusters on a 2D map produced with t-distributed stochastic neighbor embedding (t-SNE) as well as uniform manifold approximation and projection (UMAP) 79. Identification of cell types and subtypes by nonlinear dimensional reduction (t-SNE) Cells were clustered using graph-based clustering of the PCA reduced data with the Louvain Method after computing a shared nearest neighbor graph 78, 80. For sub-clustering, we applied the same procedure of scaled, dimensionality reduction, and clustering to the specific set of data (usually restricted to one type of cell.) For each cluster, we used the Wilcoxon Rank-Sum Test to find significant deferentially expressed genes comparing the remaining clusters. SingleR and known marker genes were used to identify cell type 81.

### Single-cell copy-number variation evaluation

To distinguish tumor from non-tumor cells, we evaluated levels of copy-number variation (CNV) of each cell on the chromosome by inferCNV R package (version 1.0.4) (https://github.com/broadinstitute/inferCNV). The inferCNV algorithm is based on the theoretical foundation that the relative expression of a large number of neighboring genes in the genome average out to reflect the gene-specific expression patterns and abundance distribution that primarily reflect chromosomal copy number variations (CNVs). After sorting all analyzed genes based on their genomic positions, the algorithm uses the moving average of 100 analyzed genes and the equation below to estimate the chromosome CNV for each cell and each analyzed gene: 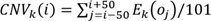. In the equation, CNV(i) represents the estimated relative copy number of cell k for the i'th gene in the sorted gene list, o_j_ is the j'th gene in the sorted gene list, and Ek(o_j_) is the relative normalized expression of that gene in cell k^82^. CNV levels of these main cell types was calculated by the amount of gene expression from the scRNA-seq data for each cell with cutoff 0.1. Genes were then sorted based on their chromosomal location and a moving average of gene expression was calculated using a window size of genes. The expression was then centered to zero by subtracting the mean. CD8+T cells were selected as reference cells, leaving all remaining cells as the observed cells. The parameters of inferCNV analysis included “denoise”, default hidden Markov model settings, and a value of 0.1 for “cutoff”.

### Simultaneous gene regulatory network analysis

SCENIC analysis was performed using the motifs database for RcisTarget and GRNboost (SCENIC, version 1.1.2.2) with default parameters. In detail, we identified transcription factor binding motifs over-represented on a gene list with RcisTarget package. The activity of each group of regulons in each cell was scored by AUCell package. To evaluate the cell type specificity of each predicted regulon, we calculated the regulon specificity score (RSS) which was based on the Jensen-Shannon divergence (JSD), a measure of the similarity between two probability distributions. Specifically, we calculated the JSD (Jensen-Shannon divergence) between each vector of binary regulon activity overlaps with the assignment of cells to a specific cell type. The connection specificity index (CSI) for all regulons was calculated with the scFunctions (https://github.com/FloWuenne/scFunctions/) package.

### Cell-cell communication analysis

Cell-cell communication analysis was conducted with the snRNA-seq data by using the cellchat package (version 1.6.1). Only receptors and ligands expressed in >10% of cells of any type from old or young group were further evaluated, while a cell-cell communication was considered nonexistent if the ligand or the receptor was unmeasurable. Averaged expression of each ligand-receptor pair was analyzed between various cell types, and only those with P value < 0. 01 were used for the prediction of cell-cell communication between any 2 cell types.

### Pseudo-time trajectory analysis

The R package monocle2 (version 2. 14. 0) was used to conduct the pseudo-time trajectory analysis 83. Briefly, a differential expression analysis was performed to identify the top significantly differentially expressed genes (FDR < 0. 05) between groups to build the disease trajectory, and then each single cell was assigned a numeric pseudo-time value and then ordered along the disease trajectory.

### Enrichment analysis and scoring

Gene Ontology (GO), and HALLMARK pathway enrichment analysis of DEGs were performed by clusterProfiler (version 3.14.0) and GSVA (version 1.48.3) package and Seurat (version 3.0.2). Part of the analysis was done with the help of Metascape^84^.

### Calculation of single-cell subgroup marker genes

We used the FindAllMarkers function in the Seurat package (version 3.0.2) to identify marker genes specific to each cell subset. To establish the marker genes, we set the threshold at Log FC > 0.25 and considered only genes with p-values < 0.05. The list of marker genes obtained from this step has been included in the supplementary materials **(Table S3)**. Subsequently, we selected genes that are functionally relevant to the subset, have available antibodies, and exhibit high expression specificity (preferably with a larger Log FC) as marker genes for immunofluorescence staining.

### ICIs RNA-Seq cohorts

To validate the predictive value of Comprehensive.sig, we systemically collected transcriptomic data and clinical information on pre-anti-PD-1 treatment samples from four SKCM cohorts (Hugo 2016 61, Liu 2019 62, Gide 2019 63, and Riaz 2017 64). We used the ComBat method to remove batch effects of different ICIs RNA-Seq cohorts 85. The patients were randomly divided into two datasets: a training set (80 %, n = 195) and a testing set (20 %, n = 49).

### Clinical outcomes

The primary clinical outcomes were ORR and overall survival (OS). ORR was assessed using Response Evaluation Criteria in Solid Tumors (RECIST) version 1.1 in all cohorts 86, except the Hugo 2016 cohort 61, whose ORR was assessed using immunorelated RECIST (irRECIST). The patients were divided into two groups according to their response status: PD and NPD.

### Model training and parameter tuning

We trained the ICIs treatment response classification model with Comprehensive.Sig, using seven common machine learning (ML) algorithms, including support vector machine (“SVM”), Naïve Bayes (“NB”), random forest (“RF”), k-nearest neighbors (“KNN”), AdaBoost Classification Trees (“AdaBoost”), boosted logistic regressions (“LogiBoost”), and cancerclass 87, 88. For each ML algorithm with parameters except cancerclass, fivefold cross-validation (CV) was adopted for hyperparameter tuning to optimize the performance of the model. To ensure robustness, we repeated the optimization process 10 times with different random seeds for each single resampling 89. As for cancerclass which does not require parameters, we trained the model using the entire training set directly.

### Model testing

Seven models were derived from the training set using different ML algorithms. These models were then applied to the test set and the results were compared. The model with the best performance was selected as the final model.

### Immunofluorescence staining

For FFPE tissue sections, heat mediated antigen retrieval was performed with citrate pH 6 in a microwave oven for 30 min after deparaffinization. After washing with PBS, tissue sections were permeabilized and blocked using a solution with TBS containing 0.3% Triton X-100 and 5% donkey serum (D-TBST solution) for1 h at room temperature. Then the tissue sections were incubated with primary antibody diluted with D-TBST solution at 4 °C overnight. After washing with TBS containing 0.05% Tween-20 for three times, the tissue sections were incubated with secondary antibody and Hochest (1:1000) diluted with D-TBST solution at room temperature for 1.5 h. After washing with TBST, slides were mounted in mounting solution, cover slipped, and sealed with nail polish. After confocal microscopy, Slideviewer software was used to observe and record the number of positive cells under high magnification(80x).

### Statistical analysis

To conduct statistical tests using R (version 4.1.1) or GraphPad Prism 7.0 (GraphPad Software, USA), the following procedures were followed. For non-paired samples, the first step involved assessing the normality of the data distribution. If the data followed a normal distribution and exhibited homogeneity of variances, the non-paired t-test was selected. In cases where the homogeneity of variances assumption was violated, the Welch's correction for the non-paired t-test was applied. For non-normally distributed data, the non-parametric Mann-Whitney rank-sum test was used. For paired samples, the initial step was to verify the normality of the differences between paired samples. If the differences followed a normal distribution and were consistent, the paired t-test was chosen. In cases where the normality assumption was violated, the ratio paired t-test was used. If the differences did not follow a normal distribution, the non-parametric Wilcoxon signed-rank test was employed. A p-value of ≤ 0.05 was considered statistically significant. A receiver operating characteristic (ROC) curve was used, and a larger AUC indicated a better predictive performance. An AUC of 0.9-1.0 is considered excellent, 0.8-0.9 very good, 0.7-0.8 good, 0.6-0.7 sufficient, 0.5-0.6 bad, and less than 0.5 considered not useful 90. Patients predicted by the final model as “PD” and “NPD” were categorized into high-risk and low-risk subgroups for survival analysis. Part of the analysis was performed using Sangerbox^91^.

## Supplementary Material

Supplementary figures and tables.

## Abbreviations

ACTA2: actin alpha 2
ACTB: actin beta
ALCAM: activated leukocyte cell adhesion molecule
anti-PD-1: PD-1 antibody
AOAH: acyloxyacyl hydrolase
AUC: area under the curve
BATF: basic leucine zipper ATF-like transcription factor
C1QA: complement C1q A chain
C1QB: complement C1q B chain
CAFs: cancer associated fibroblasts
CCL: C-C motif chemokine ligand
CCR: C-C motif chemokine receptor
CD1C: CD1c molecule
CD14: CD14 molecule
CD200: CD200 molecule
CD22: CD22 molecule
CD6: CD6 molecule
CD74: CD74 molecule
cDC1s: type 1 conventional dendritic cells
CDCA8: cell division cycle associated 8
CEBPD: CCAAT enhancer binding protein delta
CENPP: centromere protein P
CLEC: C-type lectin domain containing
CNV: copy number variation
CXCL: C-X-C motif chemokine ligand
CXCR: C-X-C motif chemokine receptor
CTLA: anti-cytotoxic T-lymphocyte associated protein
DEGs: differentially expressed genes
DLC1: DLC1 Rho GTPase activating protein
EMT: epithelial-mesenchymal transition
EZH2: enhancer of zeste 2 polycomb repressive complex 2 subunit
FCER1: Fc epsilon receptor I
FOXD3: forkhead box D3
FOXP3: forkhead box P3
GNLY: granulysin
GSVA: gene set variation analysis
GO: gene ontology
GZMA: granzyme A
HLA-DRA: major histocompatibility complex, class II, DR alpha
H&E: hematoxylin and eosin
ICIs: immune checkpoint inhibitors
ICOS: inducible T cell costimulator
IFN: interferon
IGHG1: immunoglobulin heavy constant gamma 1
IGKC: immunoglobulin kappa constant
IL2RA: interleukin 2 receptor subunit alpha
IMMP2L: inner mitochondrial membrane peptidase subunit 2
KIT: KIT proto-oncogene, receptor tyrosine kinase
KLF6: KLF transcription factor 6
KNN: k-nearest neighbors
LAG3: lymphocyte activating gene 3
MAF: MAF BZIP transcription factor
MHC: major histocompatibility complex
MKI67: marker of proliferation Ki-67
MM: malignant melanoma
MLANA: Melan-A
MMP: matrix metallopeptidase
MS4A1: membrane spanning 4-domains A1
MYO1B: myosin IB
NB: Naïve Bayes
NFIA: nuclear factor I A
NK/NKT: natural killer/natural killer T
NPD: non-progressive disease
ORR: objective response rate
PBX1: PBX homeobox 1
PD: progressive disease
PD-1: programmed cell death-1
PDCD1: programmed cell death 1
PDGFRB: platelet derived growth factor receptor beta
PTPRC: protein tyrosine phosphatase receptor type C
PROX1: prospero homeobox 1
RF: random forest
RNA-seq: RNA sequencing
RSS: regulon specificity score
ROC: receiver operating characteristic
SELL: selectin L
SKCM: skin cutaneous melanoma
snRNA-seq: single-nucleus sequencing
SOCS2: suppressor of cytokine signaling 2
SOX6: SRY(sex determining region Y)-box transcription factor 6
SPP1: secreted phosphoprotein 1
STAT: activator of transcription
SVM: support vector machine
TCGA: the Cancer Genome Atlas
TCIA: The Cancer Immunome Atlas
TCR: T cell receptor
Tef: effector CD8^+^ T cells
Tfh: follicular helper T cell
TGF-β: transforming growth factor beta
THEMIS: thymocyte-expressed molecule involved in selection
TLR4: toll like receptor 4
TME: tumor microenvironment
TNF: tumor necrosis factor
TNFSF18: TNF superfamily member 18
TOP2A: DNA topoisomerase II alpha
Tregs: regulatory T cells
TYR: tyrosinase
VIM: vimentin
VISTA: V-set immunoregulatory receptor
VWF: Von Willebrand factor
WNT: wingless-type MMTV integration site family
