# Supplementary material for: An advanced comprehensive muti-cell-type-specific model for predicting anti-PD-1 therapeutic effect in melanoma
Source: Theranostics. 2024 Mar 3;14(5):2127–50. doi: 10.7150/thno.91626 (PMC10945348; doi:10.7150/thno.91626)
Supplement: Supplementary file 1 — Supplementary figures and tables. [file thnov14p2127s1.pdf]

Supporting Information

An advanced comprehensive muti-cell-type-specific model for predicting anti-PD-1 therapeutic effect in melanoma

Wei Sun[1*] , Yu Zhu[2*], Zijian Zou[1*], Lu Wang[2], Jingqin Zhong[1], Kangjie Shen[2], Xinyi Lin[1], Zixu Gao[2], Wanlin Liu[1], Yinlam Li[2], Yu Xu[1], Ming Ren[2], Tu Hu[1], Chuanyuan Wei[2✉], Jianying Gu[2✉], Yong Chen[1✉]

[1]Department of Musculoskeletal Oncology, Fudan University Shanghai Cancer Center; Department of Oncology, Shanghai Medical College, Fudan University, Shanghai 200032, P. R. China.
[2]Department of Plastic and Reconstructive Surgery, Zhongshan Hospital, Fudan University; Cancer center, Zhongshan Hospital, Fudan University, Shanghai 200032, P. R. China.
*These authors contributed equally to this work.

✉Corresponding author. E-mail: chenyong@fudan.edu.cn; gu.jianying@zs-hospital.sh.cn; wei.chuanyuan@zs-hospital.sh.cn.

Supplemental Figure

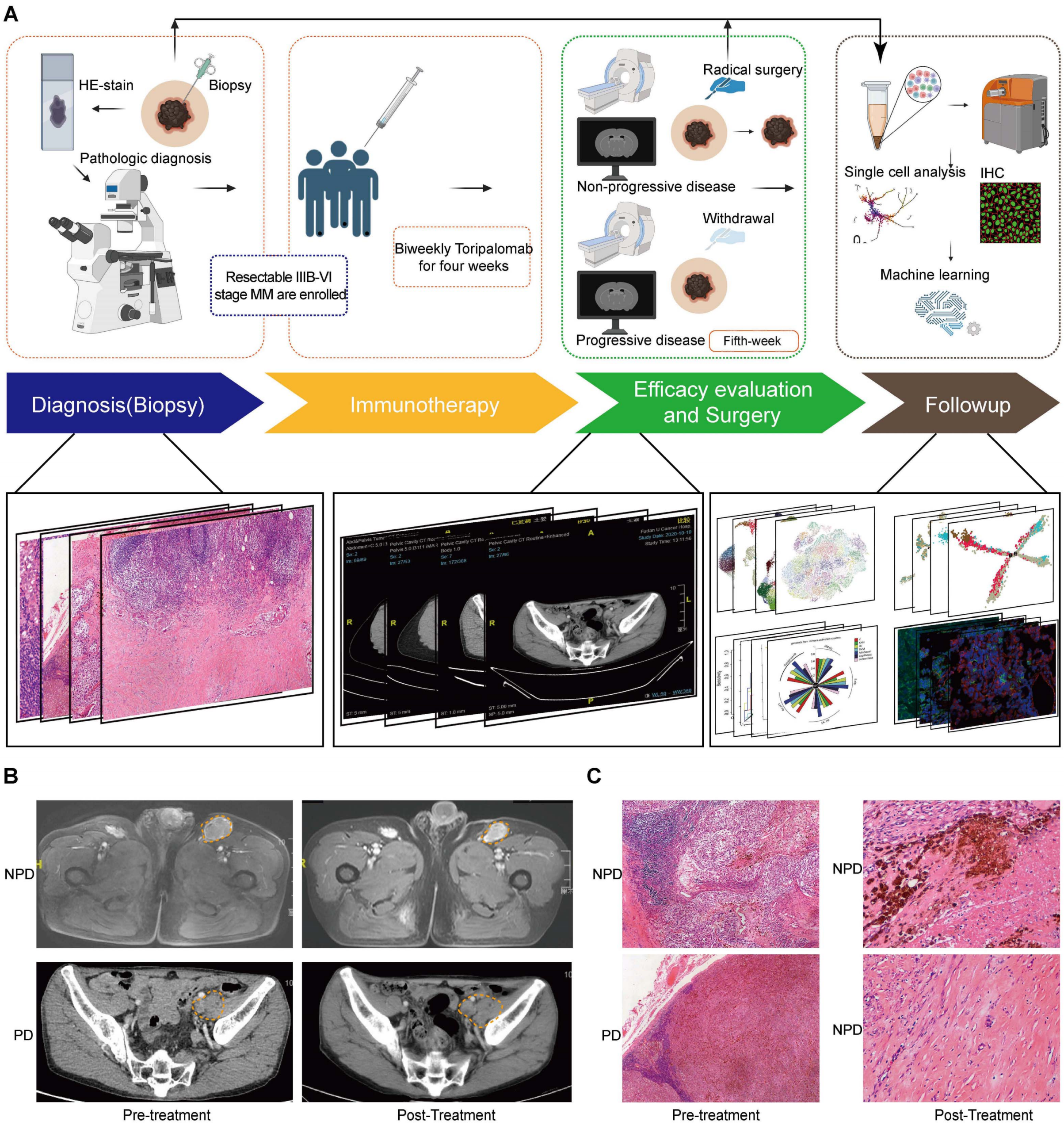

Figure S1. Schematic representation of the study A) A schematic diagram of the four stages of the study. B) Enhanced CT images of the enrolled patients for the evaluation of anti-PD-1 treatment response. C) Representative hematoxylin and eosin (H&E)-stained images of tumors in PD-pre/NPD-pre group (left) and NPD-post group (right); magnification: 40x.

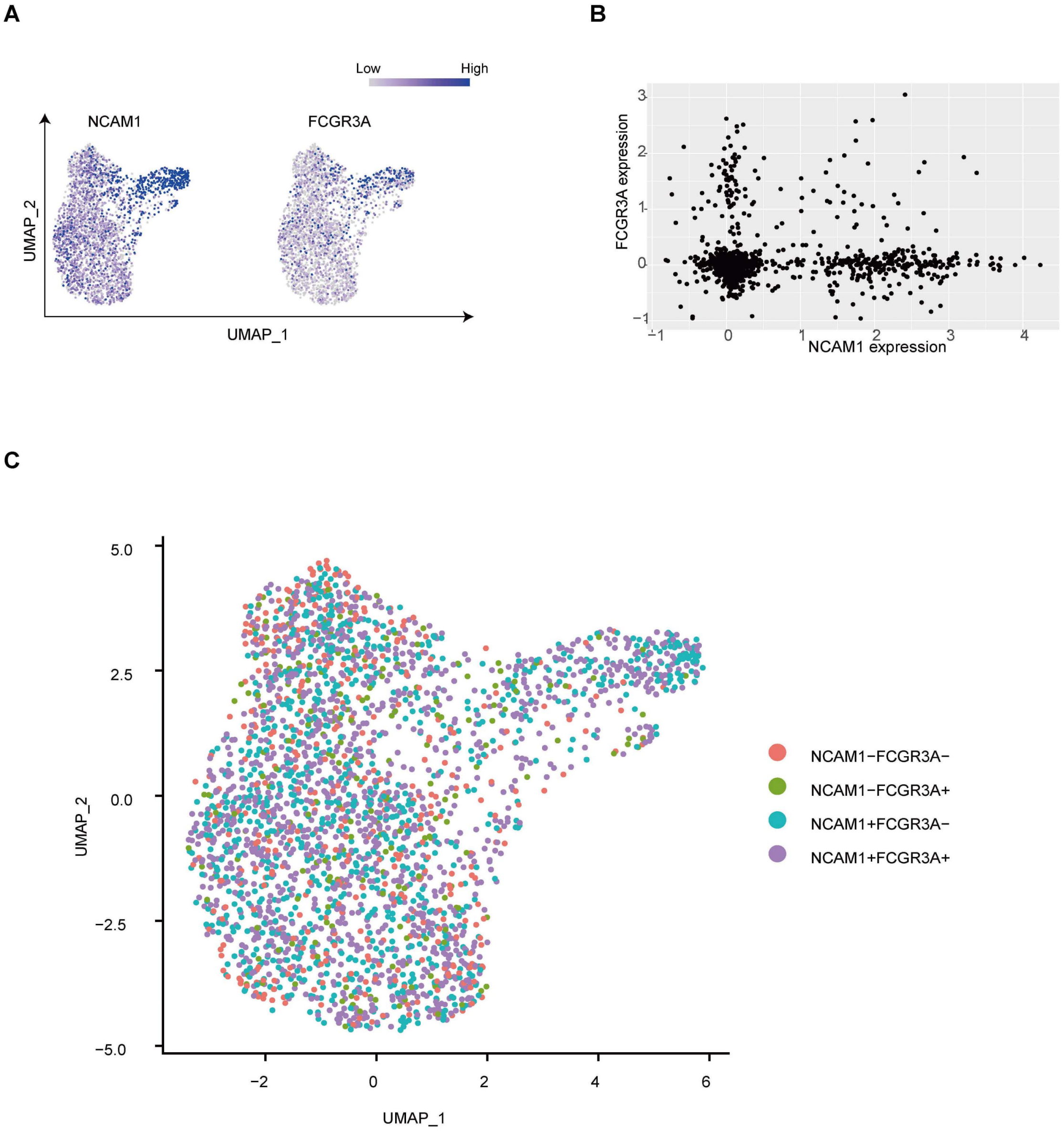

Figure S2. A. UMAP plot showing the distribution of NCAM1 and FCGR3A in NK/NKT cells **B.** Dot plot showing the distribution of NCAM1 and FCGR3A in NK cells **C.** The UMAP plot showing the distribution of NCAM1-positive and FCGR3A-positive NK/NKT cells

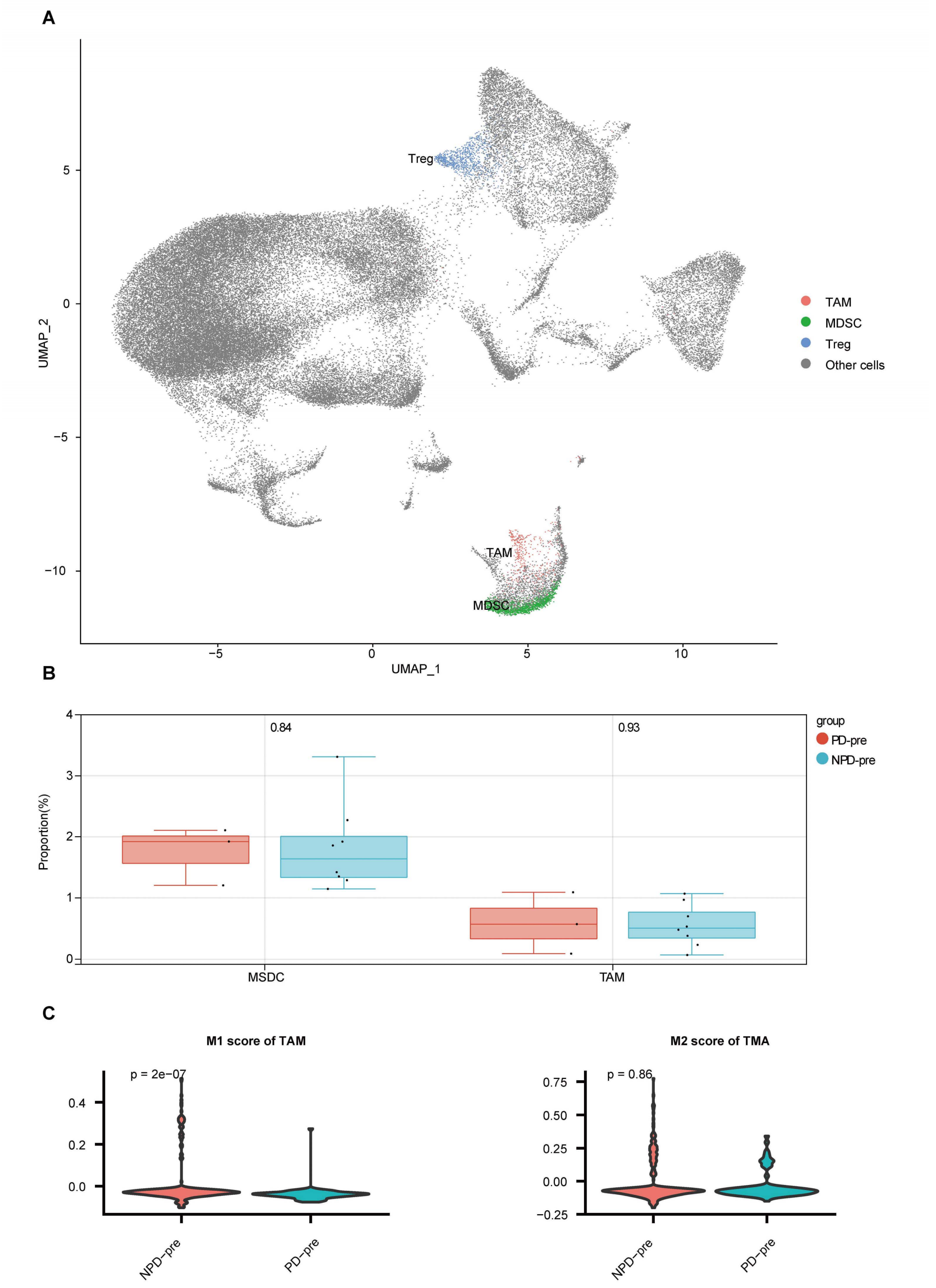

Figure S3. A. The UMAP plot showing the subgroups of Treg, TAM, and MDSC cells. **B.** Box plot showing the proportion of TAM, and MDSC between PD-pre and NPD-pre groups

(non-paired t test) **C.** Violin plots showing the differences in the M1 and M2 scores of TAM between the PD-pre and NPD-pre groups(non-paired t test).

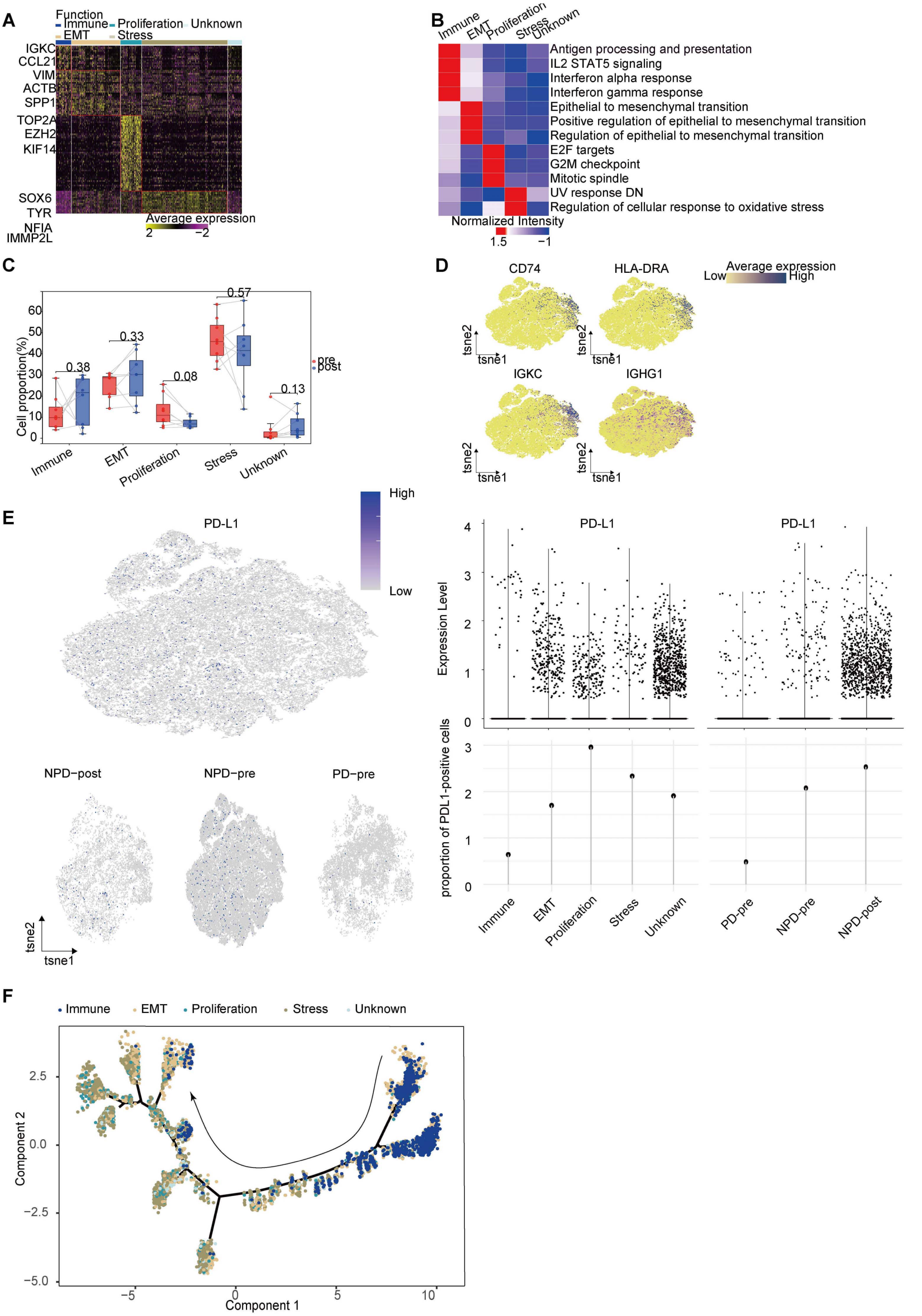

Figure S4. A. Heatmap showing the specifically highly expressed genes in each subgroup. **B.** GSVA of the GO and HALLMARK pathways in these subgroups. **C.** Box plot showing the proportion of annotated melanoma subgroups between NPD-pre and NPD-post groups (paired

t test). **D.** Feature plots showing immune related genes. **E.** The t-SNE plot and dot plot showing the distribution of PD-L1 in melanoma cells, the expression levels of PD-L1 and the proportion of PD-L1 positive cells in five melanoma subtgroups. **F.** Pseudo-temporal trajectory of NPD-pre and NPD-post melanoma cells identified two distinct cell fates colored by groups.

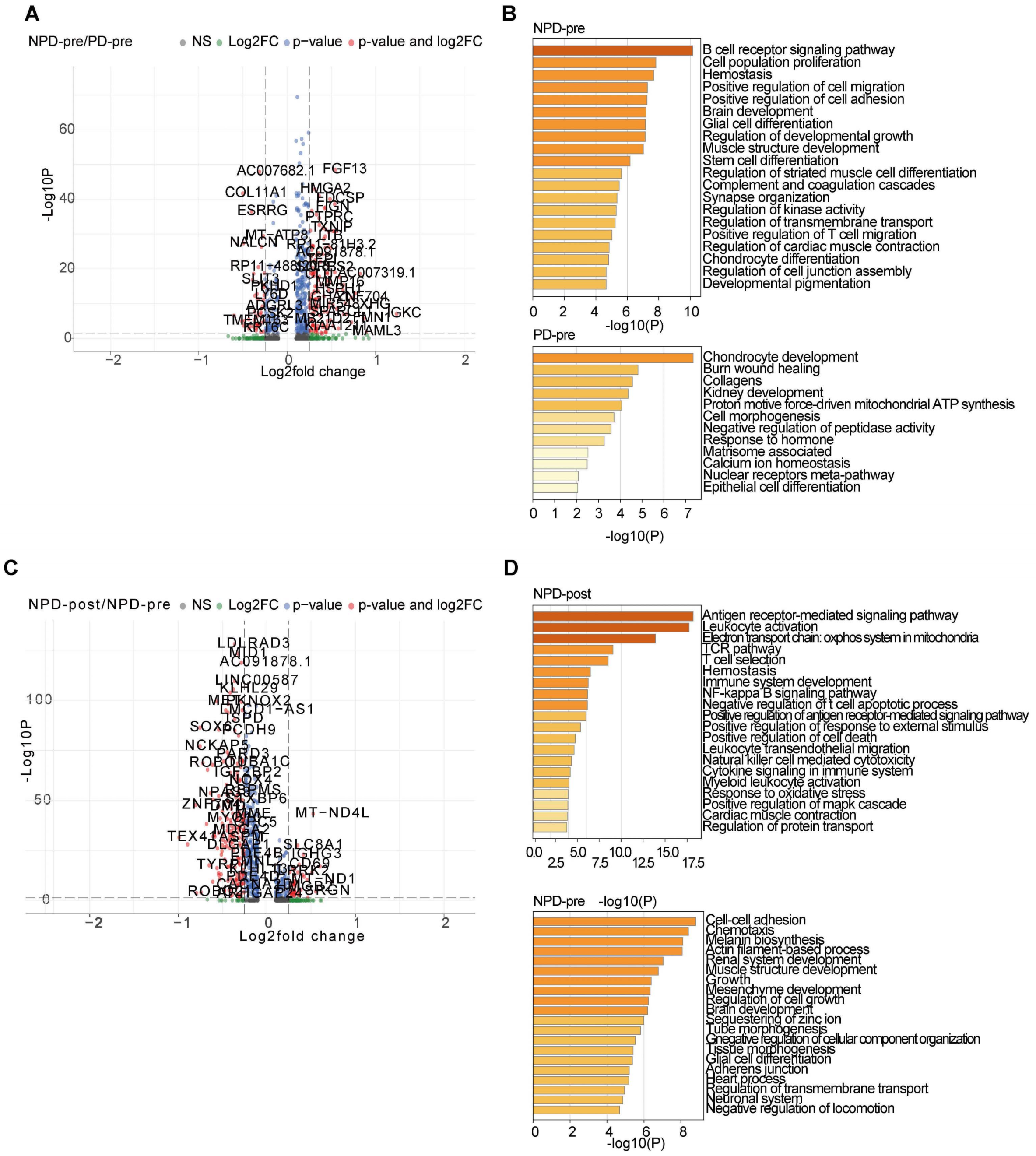

Figure S5. A. Volcano plot showing DEGs in MM_immune between PD-pre and NPD-pre groups. **B.** Bar plot showing the enriched functions for DEGs in A. **C.** Volcano plot showing DEGs in MM_immune between NPD-pre and NPD-post groups. **D.** bar plot showing the enriched functions for DEGs in C.

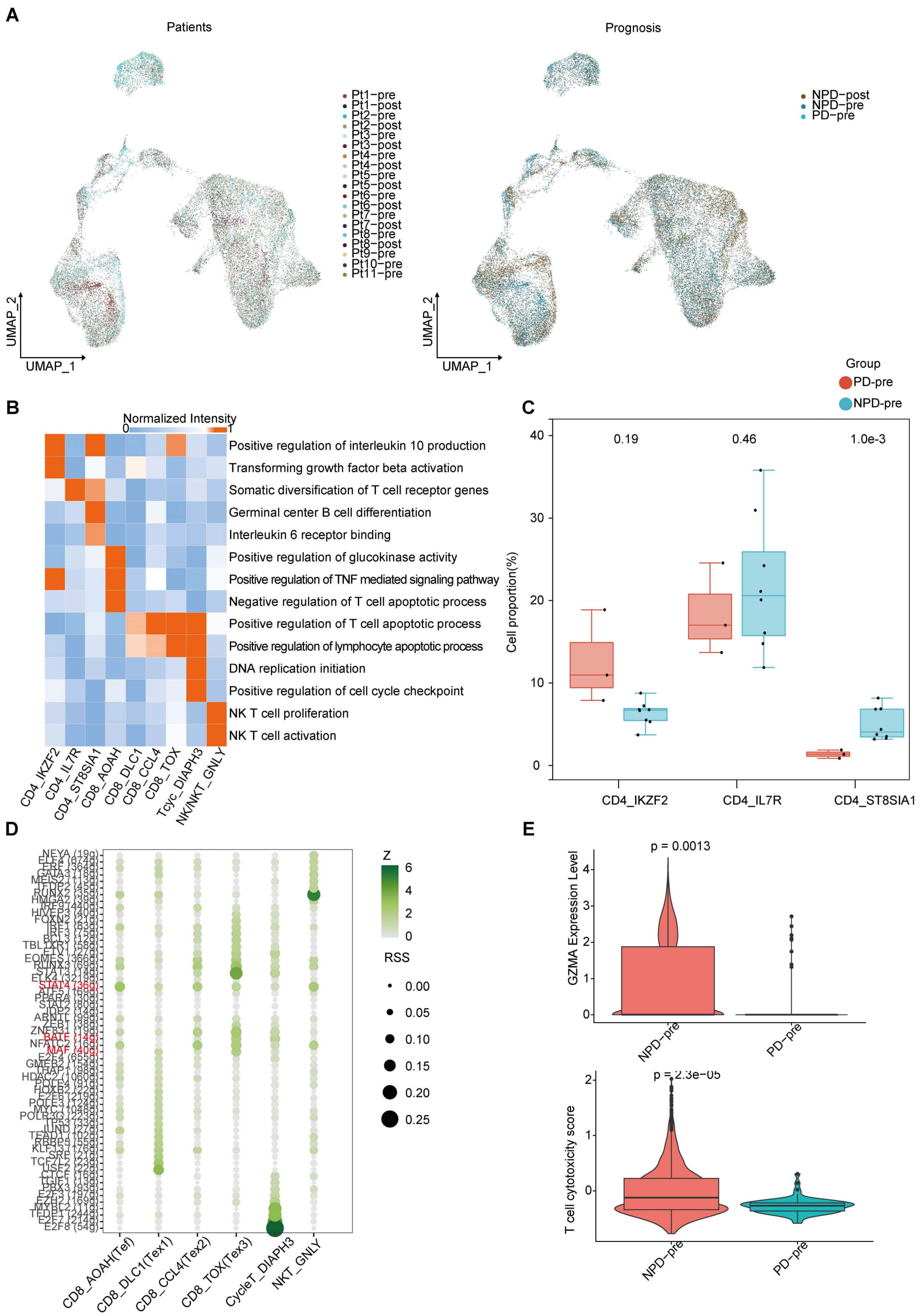

Figure S6. A. UMAP plot (left panel) of 27,766 lymphocytes, color-coded by patient origin; UMAP plot (right panel) of 27,766 lymphocytes, color-coded by groups. **B.** GSVA of the GO pathways in T cell (including NK/NKT) subgroups. **C.** Box plot showing the proportion of

annotated CD4$^+$T subgroups between PD-pre and NPD-pre groups (non-paired t test) **D.** Dot plot of the t-values of AUC scores of expression regulation by transcription factors, as estimated using SCENIC, per subgroup of CD8$^+$T and NK/NKT. **E.** Box plots and violin plots showing the differences in the expression levels of the GZMA gene and cytotoxicity scores of CD8$^+$T between the PD-pre and NPD-pre groups(non-paired t test).

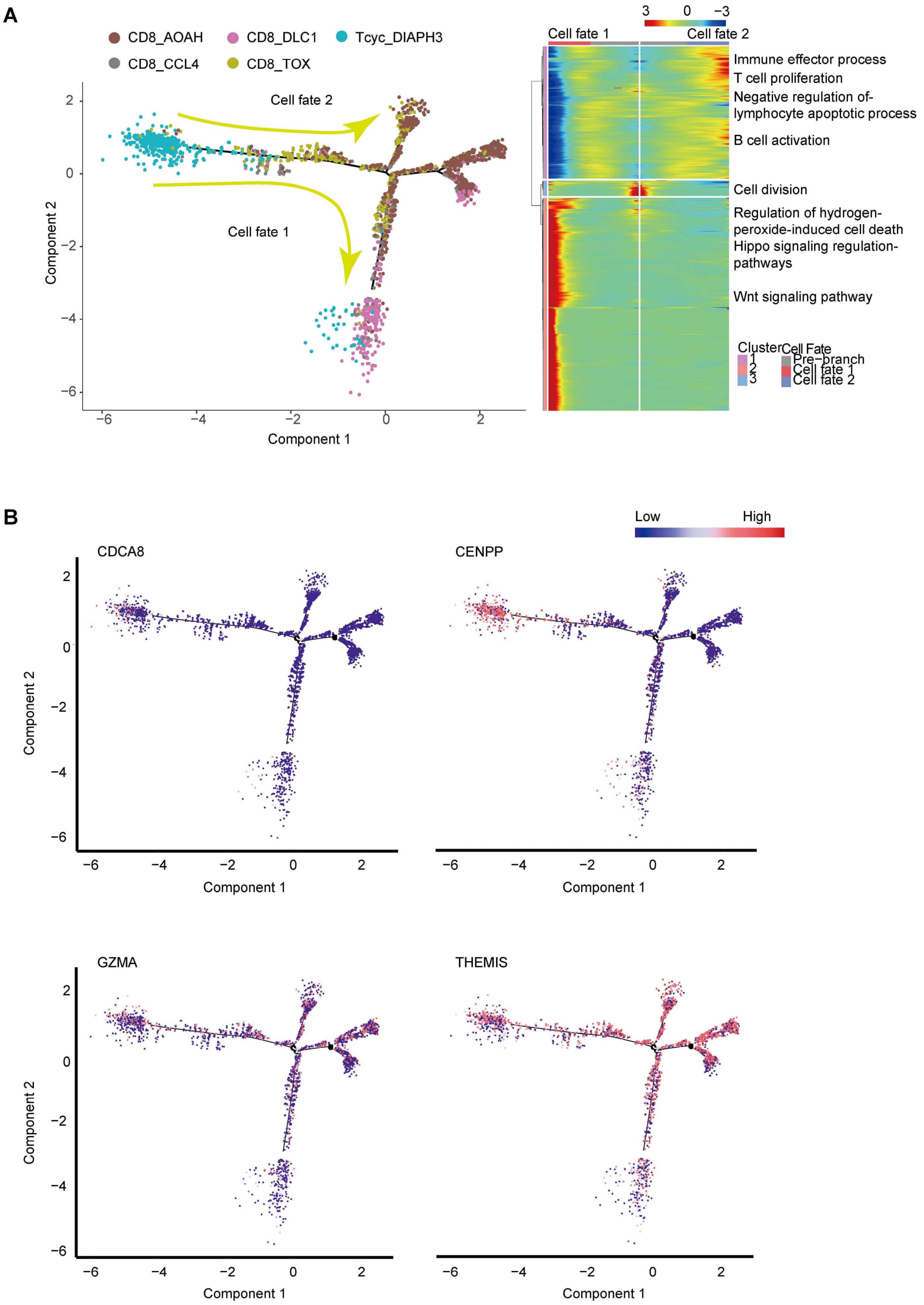

Figure S7. A. Pseudo-temporal trajectory (left panel) of CD8+T cells identified two distinct cell fates colored by subgroup; Heatmap (right panel) showing the enriched pathways in these three phases of the left panel. **B.** Pseudo-temporal trajectory of $CD8^{+}T$ cells colored by the expression of subgroup-specific genes.

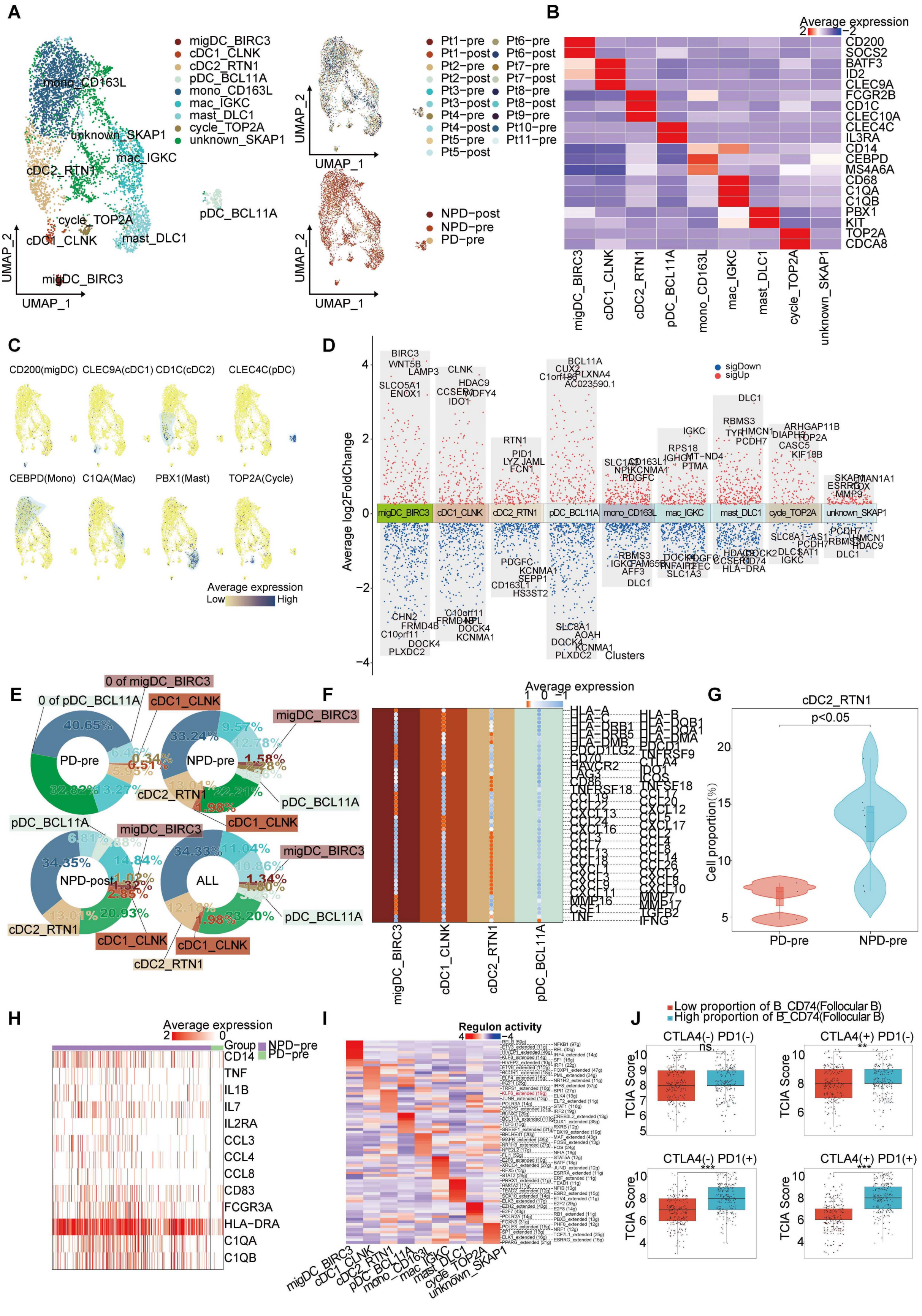

Figure S8. cDC2 showes the strongest immune activation function in myeloid cells. A. UMAP plot (left panel) of 4,999 myeloid cells, color-coded by their associated cluster; UMAP plot (upper right panel) myeloid cells, color-coded by patient origin; UMAP plot

(lower right panel) myeloid cells, color-coded by groups. **B.** Heatmap showing the specifically highly expressed genes in each myeloid cell subgroup. **C.** Heatmap the specifically highly expressed genes in each myeloid cell subgroup. **D.** Volcano plot showing the differentially expressed genes in the nine annotated subgroups. **E.** The proportion of nine annotated subgroups between groups. **F.** Expression of MHC molecule, immune checkpoint, immune regulation factor, chemokine and migration-related gene of four DC subgroups. **G.** Violin plot showing the proportion of cDC2_RTN1 between PD-pre and NPD-pre groups.(non-paired t test) **H.** Heatmap showing the expression of immune-related gene of cDC2_RTN1 between PD-pre and NPD-pre groups. **I.** Heatmap of the t-values of AUC scores of expression regulation by transcription factors, as estimated using SCENIC, per subgroup of myeloid cells. **J.** Box plots of TCIA score of TCGA patients categorized into high and low groups based on their score of the percentage of the cDC2_RTN1 subgroup.

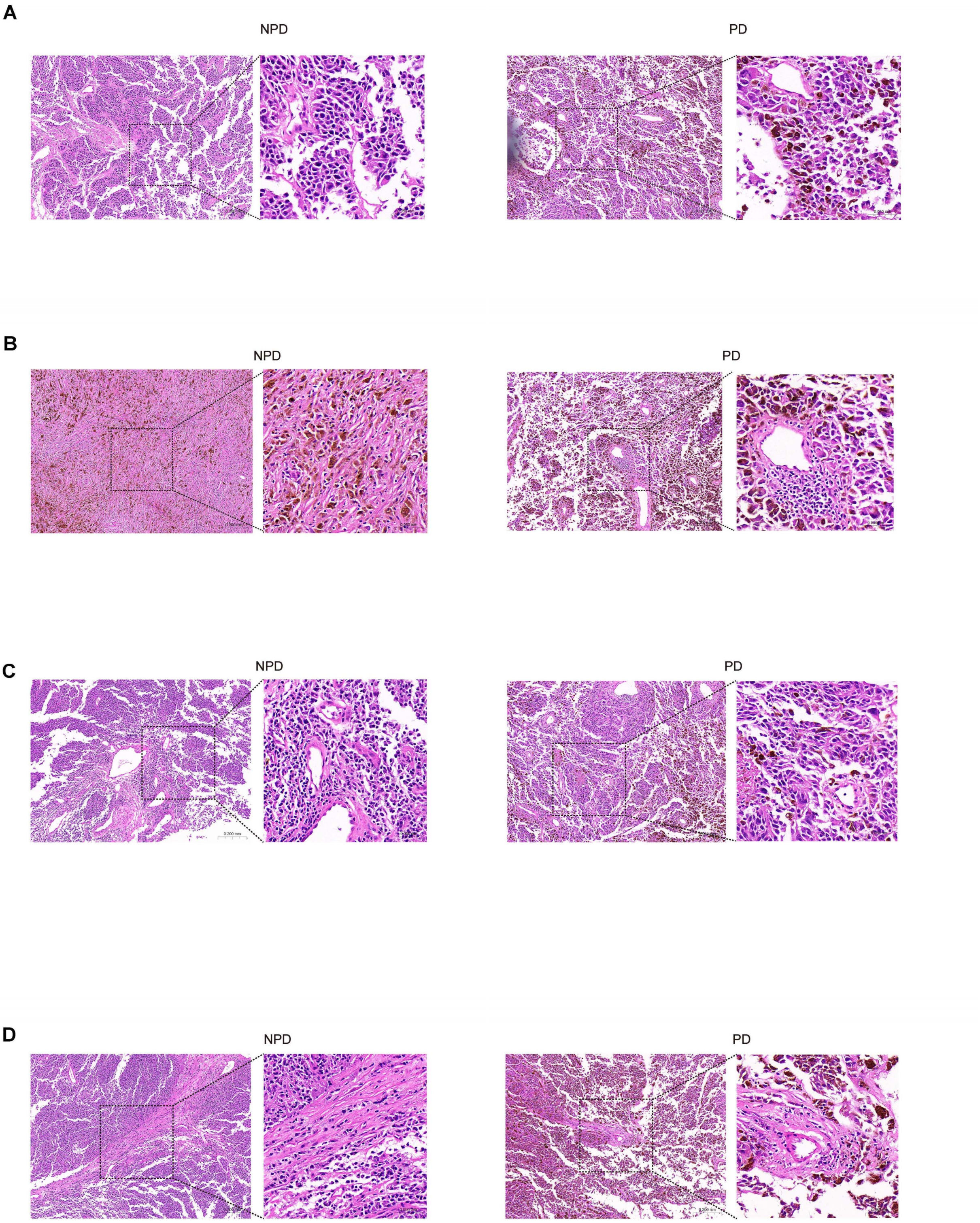

Fig. S9. A-D. Representative corresponding hematoxylin and eosin (H&E)-stained images for Fig. 6, scale bar: 200 μm (left); 50 μm (right);

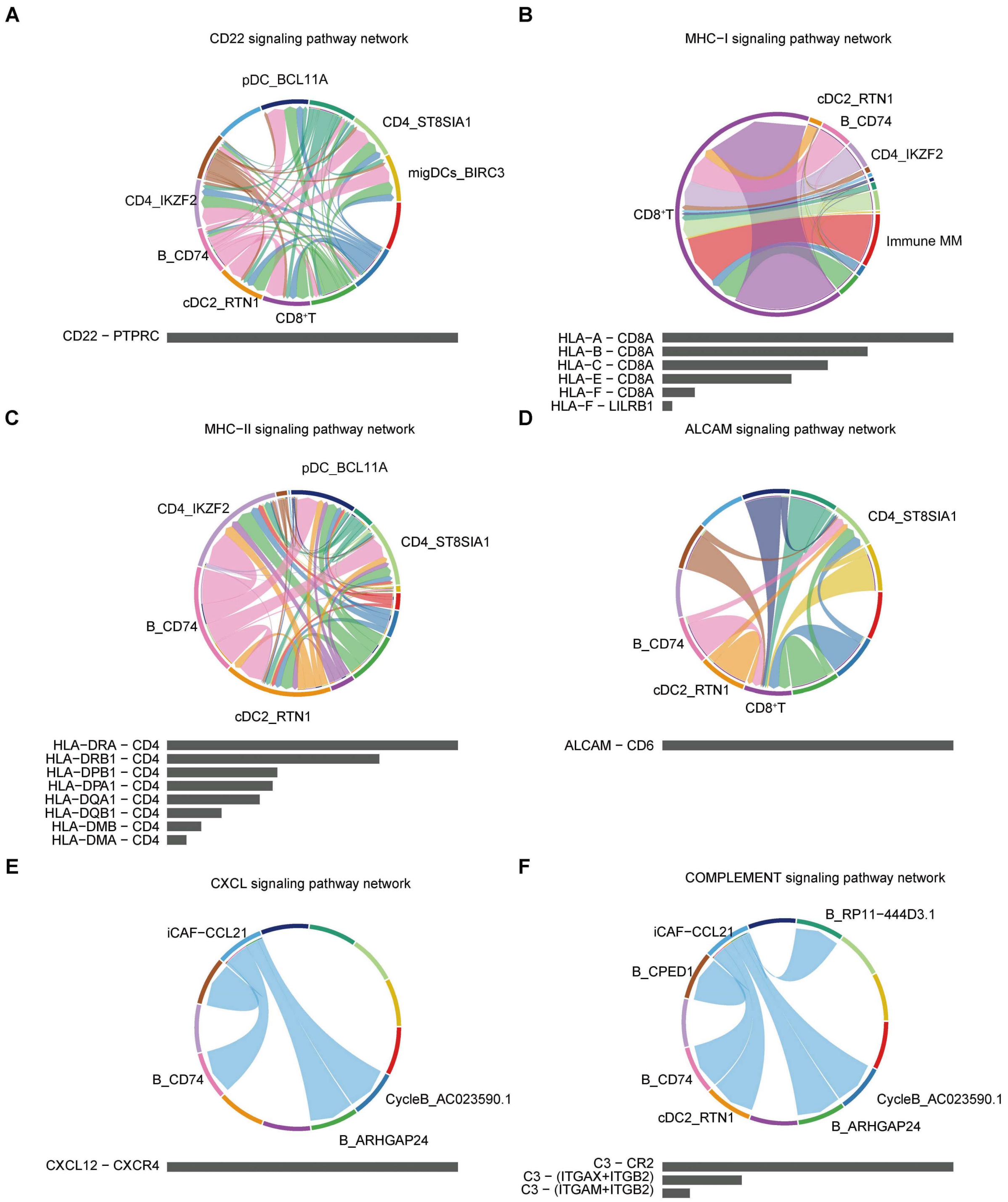

Figure S10. A-F. Chord plots showing the networks of CD22 (A), MHC−I (B), MHC−II (C), ALCAM(D), CXCL (E) and Complement (F) signaling pathways in the ecosystem of NPD-pre group.

A

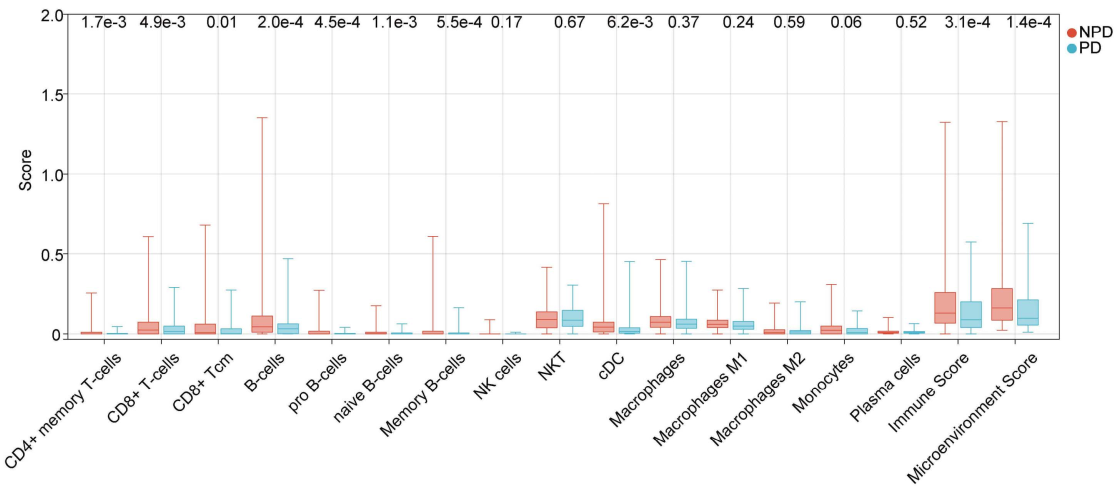

Figure S11. A. Boxplot showing the proportion the proportions of immune cells predicted by xCell for the "PD-predicted" and "NPD- predicted" groups (non-paired t-test).

Supplemental Table

Table S1. Prognostic information of patients

**Patient name**	**subject name**	**Accession number**	**Residual tumor % (total viable tumor/total surface area of cross-sectional tumor bed assessed)**	**Tumor**
CXF	Pt1	2020-40530	pNR：88%	Acral melanoma
FZD	Pt2	2020-49338	pPR:43%	Cutaneous melanoma
LYH	Pt3	2020-40591	pNR：93%	Cutaneous melanoma
LYZ	Pt4	2022-12575	pPR:36%	Cutaneous melanoma
QRJ	Pt5	2020-42505	pNR:72%	Acral melanoma
XGS	Pt6	2021-61640	pPR:37%	Cutaneous melanoma
YZY	Pt7	2020-42038	pNR：73%	Acral melanoma
ZYC	Pt8	2020-52864	pPR:25%	Acral melanoma
LSX	Pt9	2020-33375	PD: >100%	Acral melanoma
YYX	Pt10	2021-30008	PD: >100%	Cutaneous melanoma
ZWX	Pt11	2022-02343	PD: >100%	Cutaneous melanoma

Table S2. Information about the patients

submitter_id.samples	age_at_initial_pathologic_diagnosis	breslow_level	breslow_depth_value(mm)	melanoma_ulceration_indicator	neoadjuvant_medicine	melanoma_clark_level	pathologic_M
Pt1	50	3	3.1	yes	Toripalimab	4	x
Pt2	69	5	6	yes	Toripalimab	5	x
Pt4	44	5	>4.0	no	Toripalimab	NA	x
Pt5	72	NA	NA	NA	Toripalimab	NA	x
Pt6	72	NA	NA	NA	Toripalimab	NA	x
Pt7	68	5	13	yes	Toripalimab	4	x
Pt8	59	3	2.2	yes	Toripalimab	3	x
Pt9	64	4	3.7	no	Toripalimab	4	x
Pt10	68	5	>4.0	yes	Toripalimab	4	x
Pt11	56	5	5	yes	Toripalimab	5	x

submitter_id.samples	pathologic_N	pathologic_T	radiation_therapy	submitted_tumor_location	tissue_source_site	withdrawn	year_of_initial_pathologic_diagnosis
Pt1	0	3b	no	left popliteal fossa	lymph node	no	2017/5/1
Pt2	3b	4b	no	left groin	lymph node	no	2020/11/25
Pt4	3b	4a	no	right axilla	lymph node	no	2020/7/27
Pt5	3b	x	no	right groin	lymph node	no	2020/1/1
Pt6	1a	x	no	left axilla	lymph node	no	2020/3/6
Pt7	3b	4b	no	right groin	lymph node	no	2020/9/9
Pt8	3b	3b	no	right popliteal fossa	lymph node	no	2019/10/15
Pt9	1a	3b	no	left groin	lymph node	yes	2020/8/1

Pt10	0	4b	no	right axilla	lymph node	yes	2020/4/20
Pt11	3b	4b	no	right groin	lymph node	yes	2022/1/29

submitter_id.samples	gender.demographic	vital_status.demographic	year_of_death.demographic	age_at_diagnosis.diagnoses	prior_malignancy.diagnoses	tumor_stage.diagnoses	year_of_diagnosis.diagnoses
Pt1	female	alive	NA	50	melanoma	2b	2017/5/1
Pt2	male	dead	2022/3/1	69	melanoma	3d	2020/11/25
Pt4	female	alive	NA	44	melanoma	3d	2020/8/13
Pt5	female	dead	2021/11/2	72	melanoma	x	2020/1/1
Pt6	male	alive	NA	72	melanoma	x	2020/3/6
Pt7	female	alive	NA	68	melanoma	3d	2020/9/9
Pt8	male	alive	NA	59	melanoma	3c	2019/10/15
Pt9	male	dead	2020/10/9	64	melanoma	3c	2020/9/11
Pt10	male	alive	NA	68	melanoma	2c	2020/5/22
Pt11	male	alive	NA	56	melanoma	3d	2022/1/29

submitter_id.samples	disease_type	nerve_invasion	Vascular_invasion	Number_of_(+)SLN	total_number_of_sentinel_lymph_nodes	Number_of_(+)NSLN	OS/month
Pt1	acral melanoma	no	yes	NA	NA	5	15
Pt2	acral melanoma	yes	yes	1	1	5	63
Pt4	cutaneous melanoma	NA	NA	1	1	1	36
Pt5	acral melanoma	NA	NA	2	2	10	22

Pt6	cutaneous melanoma	no	yes	1	1	8	35
Pt7	acral melanoma	no	no	1	1	NA	2
Pt8	acral melanoma	NA	NA	0	13	1	39
Pt9	acral melanoma	NA	NA	NA	NA	5	1
Pt10	cutaneous melanoma	NA	NA	1	1	0	40
Pt11	acral melanoma	NA	NA	4	4	1	45

Table S3. Markers for the MM_Immune, B_CD74, cDC2_RTN1, and iCAF-CCL21 subgroups

Subgroup	Marker
MM_Immune	S100A11
MM_Immune	FTL
MM_Immune	RPS21
MM_Immune	UBC
MM_Immune	TMSB10
MM_Immune	PTMA
MM_Immune	S100A6
MM_Immune	H2AFZ
MM_Immune	VIM
MM_Immune	IGKC
MM_Immune	FTH1
MM_Immune	LGALS1
MM_Immune	MT-ND5
MM_Immune	CRYAB
MM_Immune	MT-ND2
MM_Immune	S100A1
MM_Immune	RPS18
MM_Immune	MT2A
MM_Immune	MT-CO3
MM_Immune	ATP5E
MM_Immune	MT-ND4L
MM_Immune	MT-ND4
MM_Immune	MT-ND3
MM_Immune	MT-ND1
MM_Immune	ACTB
MM_Immune	MT-ATP6
MM_Immune	MT-CO1
MM_Immune	S100A10
MM_Immune	MT-CYB
MM_Immune	MT-CO2
MM_Immune	HSPB1
MM_Immune	SPARC
MM_Immune	APOC1
MM_Immune	CSTB
MM_Immune	IGHG4
MM_Immune	SPP1
MM_Immune	IGHG3
MM_Immune	IGFBP2
MM_Immune	IGLC2
MM_Immune	IGHG1
MM_Immune	IGFBP7
MM_Immune	SERPINE2
MM_Immune	S100A4
MM_Immune	ANXA1

MM_Immune	RP11-191L9.4
MM_Immune	CCND1
MM_Immune	MT1E
MM_Immune	IGLC3
MM_Immune	ARL6IP1
MM_Immune	PTPRZ1
MM_Immune	MT-ATP8
MM_Immune	C11orf96
MM_Immune	FABP5
MM_Immune	PMEL
MM_Immune	EDNRB
MM_Immune	CTSB
MM_Immune	MGP
MM_Immune	ABCB5
MM_Immune	RP11-30J20.1
MM_Immune	KRT14
MM_Immune	TYRP1
MM_Immune	GDF15
MM_Immune	LYZ
MM_Immune	C1QB
MM_Immune	AC007319.1
MM_Immune	NELL1
MM_Immune	RP11-317N12.1
MM_Immune	C1QA
MM_Immune	TGFBI
MM_Immune	S100A9
MM_Immune	MT-ND6
MM_Immune	JCHAIN
MM_Immune	HIST1H4C
MM_Immune	S100A8
MM_Immune	CCL21
MM_Immune	SPARCL1
MM_Immune	APP
MM_Immune	CLU
MM_Immune	PTGDS
MM_Immune	CXCL10
MM_Immune	FRMD5
MM_Immune	RP11-734I18.1
MM_Immune	WWTR1
MM_Immune	HLA-DRA
MM_Immune	RP11-608O21.1
MM_Immune	LINGO2
MM_Immune	MIR548XHG
MM_Immune	IGHGP
MM_Immune	RHOBTB3

MM_Immune	A2M
MM_Immune	DCT
MM_Immune	ROBO2
MM_Immune	NOV
MM_Immune	IL1RAPL1
MM_Immune	KPNA2
MM_Immune	ITGA11
MM_Immune	LRRTM4
MM_Immune	XBP1
MM_Immune	CD14
MM_Immune	POLR2F
MM_Immune	POSTN
MM_Immune	HES1
MM_Immune	KRT6A
iCAF-CCL21	CACNA2D3
iCAF-CCL21	FDCSP
iCAF-CCL21	XKR4
iCAF-CCL21	CCL21
iCAF-CCL21	LIFR
iCAF-CCL21	ADAM12
iCAF-CCL21	CLU
iCAF-CCL21	ZFPM2
iCAF-CCL21	SDK1
iCAF-CCL21	TENM3
iCAF-CCL21	ABI3BP
iCAF-CCL21	ROBO1
iCAF-CCL21	ANKRD29
iCAF-CCL21	SLC22A3
iCAF-CCL21	C7
iCAF-CCL21	LPAR1
iCAF-CCL21	C3
iCAF-CCL21	ABCC3
iCAF-CCL21	VCAM1
iCAF-CCL21	GRIN2B
iCAF-CCL21	SEMA5A
iCAF-CCL21	LAMA3
iCAF-CCL21	TMEM178B
iCAF-CCL21	OSMR
iCAF-CCL21	SYNPO2
iCAF-CCL21	CDH11
iCAF-CCL21	RNF150
iCAF-CCL21	NR2F2-AS1
iCAF-CCL21	HS3ST3A1
iCAF-CCL21	SNAP91
iCAF-CCL21	NCEH1
iCAF-CCL21	NOVA1
iCAF-CCL21	RORA
iCAF-CCL21	ST6GALNAC5

iCAF-CCL21	SLCO3A1
iCAF-CCL21	ADAMTS9-AS2
iCAF-CCL21	GNA14
iCAF-CCL21	FAM155A
iCAF-CCL21	PARD3B
iCAF-CCL21	CCL19
iCAF-CCL21	CACNA1C
iCAF-CCL21	CR2
iCAF-CCL21	PRRX1
iCAF-CCL21	RP1-84O15.2
iCAF-CCL21	PAPPA
iCAF-CCL21	NRXN3
iCAF-CCL21	KIAA1217
iCAF-CCL21	CXCL14
iCAF-CCL21	SVIL
iCAF-CCL21	BICC1
iCAF-CCL21	VEGFC
iCAF-CCL21	SLC9A9
iCAF-CCL21	CCDC102B
iCAF-CCL21	ALPK2
iCAF-CCL21	CLMP
iCAF-CCL21	DLG2
iCAF-CCL21	ZNF804B
iCAF-CCL21	FLRT2
iCAF-CCL21	MAGI1
iCAF-CCL21	NOL4
iCAF-CCL21	EPB41L2
iCAF-CCL21	NFASC
iCAF-CCL21	NFIB
iCAF-CCL21	SLIT3
iCAF-CCL21	SVEP1
iCAF-CCL21	NRG1
iCAF-CCL21	IGFBP3
iCAF-CCL21	ARHGAP29
iCAF-CCL21	TNFAIP3
iCAF-CCL21	LINC01197
iCAF-CCL21	NAV2
iCAF-CCL21	PTGDS
iCAF-CCL21	ADGRL2
iCAF-CCL21	SLIT2
iCAF-CCL21	NKAIN2
iCAF-CCL21	RARB
iCAF-CCL21	ROBO2
iCAF-CCL21	TENM4
iCAF-CCL21	IL15
iCAF-CCL21	COL4A4
iCAF-CCL21	PRUNE2
iCAF-CCL21	SEMA3A

iCAF-CCL21	DTNA
iCAF-CCL21	EPHA3
iCAF-CCL21	TMEM108
iCAF-CCL21	ARHGAP42
iCAF-CCL21	IGFBP7
iCAF-CCL21	CHL1
iCAF-CCL21	CACNA2D1
iCAF-CCL21	MAST4
iCAF-CCL21	PTPRQ
iCAF-CCL21	EXOC3L4
iCAF-CCL21	TNS3
iCAF-CCL21	CHN1
iCAF-CCL21	PTPRT
iCAF-CCL21	EBF3
iCAF-CCL21	PAPSS2
iCAF-CCL21	TPM1
iCAF-CCL21	CXCL13
iCAF-CCL21	KLHL29
iCAF-CCL21	SYNDIG1
iCAF-CCL21	RBPMS
iCAF-CCL21	COL6A3
iCAF-CCL21	IL33
iCAF-CCL21	GABRG3
iCAF-CCL21	RAI14
iCAF-CCL21	SLC26A7
iCAF-CCL21	PLD1
iCAF-CCL21	FGF14
iCAF-CCL21	KIRREL3
iCAF-CCL21	CALD1
iCAF-CCL21	NCKAP5
iCAF-CCL21	EFNA5
iCAF-CCL21	GFRA1
iCAF-CCL21	FGF7
iCAF-CCL21	ALDH1A2
iCAF-CCL21	MACROD2
iCAF-CCL21	LSAMP
iCAF-CCL21	ITGA1
iCAF-CCL21	IL7
iCAF-CCL21	ACSS3
iCAF-CCL21	GNA14-AS1
iCAF-CCL21	KCNE4
iCAF-CCL21	PID1
iCAF-CCL21	ENOX1
iCAF-CCL21	FHOD3
iCAF-CCL21	KALRN
iCAF-CCL21	TNC
iCAF-CCL21	NAALADL2
iCAF-CCL21	LIN7A
iCAF-CCL21	CRIM1

iCAF-CCL21	PLPP3
iCAF-CCL21	PALLD
iCAF-CCL21	ANK2
iCAF-CCL21	EGFR
iCAF-CCL21	PLD5
iCAF-CCL21	LURAP1L
iCAF-CCL21	PLEKHG1
iCAF-CCL21	PCDH11X
iCAF-CCL21	COL12A1
iCAF-CCL21	PTPRK
iCAF-CCL21	LINC00693
iCAF-CCL21	SLC24A3
iCAF-CCL21	HEG1
iCAF-CCL21	SLC1A2
iCAF-CCL21	SULF1
iCAF-CCL21	GLIS3
iCAF-CCL21	ITIH5
iCAF-CCL21	USP53
iCAF-CCL21	ADAMTS3
iCAF-CCL21	DCLK1
iCAF-CCL21	MYLK
iCAF-CCL21	FBXL7
iCAF-CCL21	SLCO2B1
iCAF-CCL21	CFH
iCAF-CCL21	APBB2
iCAF-CCL21	TCF4
iCAF-CCL21	CSMD1
iCAF-CCL21	FGD6
iCAF-CCL21	CXCL12
iCAF-CCL21	TBX15
iCAF-CCL21	PLA2G4C
iCAF-CCL21	PTPRM
iCAF-CCL21	MUSK
iCAF-CCL21	LTBP1
iCAF-CCL21	LARGE
iCAF-CCL21	TNFRSF11B
iCAF-CCL21	C1R
iCAF-CCL21	IRAK3
iCAF-CCL21	CR1
iCAF-CCL21	GHR
iCAF-CCL21	SLCO1A2
iCAF-CCL21	DSE
iCAF-CCL21	SDC2
iCAF-CCL21	ARHGAP24
iCAF-CCL21	CNTN1
iCAF-CCL21	RP11-266O8.1
iCAF-CCL21	DDR2
iCAF-CCL21	NR2F2

iCAF-CCL21	LRRC4C
iCAF-CCL21	ARNTL2
iCAF-CCL21	FBN1
iCAF-CCL21	EPAS1
iCAF-CCL21	RAMP3
iCAF-CCL21	RP11-81H14.2
iCAF-CCL21	RFTN1
iCAF-CCL21	COL5A2
iCAF-CCL21	NR2F1-AS1
iCAF-CCL21	THRB
iCAF-CCL21	RBMS3
iCAF-CCL21	RAPGEF5
iCAF-CCL21	MIR4435-2HG
iCAF-CCL21	PBX1
iCAF-CCL21	MROH9
iCAF-CCL21	GUCY1A3
iCAF-CCL21	PLXNA4
iCAF-CCL21	FNDC1
iCAF-CCL21	COL4A2
iCAF-CCL21	COL6A2
iCAF-CCL21	CDK14
iCAF-CCL21	DAPK1
iCAF-CCL21	ACTA2
iCAF-CCL21	GUCY1B3
iCAF-CCL21	MEOX2
iCAF-CCL21	TCF7L2
iCAF-CCL21	RP11-739G5.1
iCAF-CCL21	C10orf11
iCAF-CCL21	RIN2
iCAF-CCL21	ITPR2
iCAF-CCL21	ANKS1B
iCAF-CCL21	PCOLCE2
iCAF-CCL21	NNMT
iCAF-CCL21	SRGAP1
iCAF-CCL21	ADAMTS12
iCAF-CCL21	ADAMTS9
iCAF-CCL21	COL6A1
iCAF-CCL21	TFPI
iCAF-CCL21	FAM20A
iCAF-CCL21	RUNX1T1
iCAF-CCL21	ZNF804A
iCAF-CCL21	UNC5C
iCAF-CCL21	LHFP
iCAF-CCL21	ANTXR1
iCAF-CCL21	FIGN
iCAF-CCL21	SMOC2

iCAF-CCL21	CLSTN2
iCAF-CCL21	DEPTOR
iCAF-CCL21	ABTB2
iCAF-CCL21	COL1A2
iCAF-CCL21	NRG2
iCAF-CCL21	NEGR1
iCAF-CCL21	PDGFRB
iCAF-CCL21	GRIA4
iCAF-CCL21	CPE
iCAF-CCL21	ZNF385B
iCAF-CCL21	DPYSL3
iCAF-CCL21	PKP2
iCAF-CCL21	ZNF521
iCAF-CCL21	OCA2
iCAF-CCL21	GUCY1A2
iCAF-CCL21	PARM1
iCAF-CCL21	KIAA1211
iCAF-CCL21	NFIA
iCAF-CCL21	SORCS1
iCAF-CCL21	FNDC3B
iCAF-CCL21	GREM1
iCAF-CCL21	RSPO3
iCAF-CCL21	AFAP1
iCAF-CCL21	SLC8A1
iCAF-CCL21	IER3
iCAF-CCL21	MYO16
iCAF-CCL21	ZBTB46
iCAF-CCL21	GEM
iCAF-CCL21	KCNT2
iCAF-CCL21	VSTM4
iCAF-CCL21	CARMN
iCAF-CCL21	ELN
iCAF-CCL21	COL18A1
iCAF-CCL21	ERG
iCAF-CCL21	LINC01482
iCAF-CCL21	CSRNP3
iCAF-CCL21	LINC01239
iCAF-CCL21	NLGN4Y
iCAF-CCL21	CDH1
iCAF-CCL21	PIEZO2
iCAF-CCL21	DOK6
iCAF-CCL21	RP11-759A24.3
iCAF-CCL21	COL1A1
iCAF-CCL21	DCBLD2
iCAF-CCL21	GPC6
iCAF-CCL21	NPHS1
iCAF-CCL21	TIMP1
iCAF-CCL21	PAK3

iCAF-CCL21	CHST15
iCAF-CCL21	OLFML2B
iCAF-CCL21	EPB41L4A
iCAF-CCL21	VEGFA
iCAF-CCL21	ADGRL3
iCAF-CCL21	G0S2
iCAF-CCL21	COL23A1
iCAF-CCL21	ABCA1
iCAF-CCL21	CNTN4
iCAF-CCL21	COLEC12
iCAF-CCL21	ZFHX4
iCAF-CCL21	COL14A1
iCAF-CCL21	EPSTI1
iCAF-CCL21	RP11-64D24.2
iCAF-CCL21	GXYLT2
iCAF-CCL21	COL3A1
iCAF-CCL21	GPR176
iCAF-CCL21	NCALD
iCAF-CCL21	CADPS2
iCAF-CCL21	PDLIM3
iCAF-CCL21	PTPN13
iCAF-CCL21	LINC01524
iCAF-CCL21	TAGLN
iCAF-CCL21	LTBP2
iCAF-CCL21	TTC28
iCAF-CCL21	NLGN4X
iCAF-CCL21	ABLIM3
iCAF-CCL21	COL4A1
iCAF-CCL21	TMEM132C
iCAF-CCL21	PAM
iCAF-CCL21	NHS
iCAF-CCL21	ABLIM1
iCAF-CCL21	MYO1E
iCAF-CCL21	ME3
iCAF-CCL21	DDAH1
iCAF-CCL21	ZNF423
iCAF-CCL21	FILIP1
iCAF-CCL21	CDH2
iCAF-CCL21	FMO2
iCAF-CCL21	ID3
iCAF-CCL21	FSTL1
iCAF-CCL21	VIM
iCAF-CCL21	EFNB2
iCAF-CCL21	PLCL1
iCAF-CCL21	FAM46A
iCAF-CCL21	CCL2
iCAF-CCL21	PLSCR4
iCAF-CCL21	LRRK1

iCAF-CCL21	ETS2
iCAF-CCL21	TTLL7
iCAF-CCL21	MSRB3
iCAF-CCL21	MAPK10
iCAF-CCL21	TSHZ2
iCAF-CCL21	EPB41L3
iCAF-CCL21	SMAD1
iCAF-CCL21	ADAMTSL3
iCAF-CCL21	FAM171A1
iCAF-CCL21	TCF7L1
iCAF-CCL21	LDLRAD4
iCAF-CCL21	VGLL3
iCAF-CCL21	MYO1B
iCAF-CCL21	ST8SIA1
iCAF-CCL21	LUM
iCAF-CCL21	IL1R1
iCAF-CCL21	ADRA1A
iCAF-CCL21	CYP1B1-AS1
iCAF-CCL21	EGFLAM
iCAF-CCL21	SLCO2A1
iCAF-CCL21	ARHGAP26
iCAF-CCL21	ANOS1
iCAF-CCL21	TNFAIP2
iCAF-CCL21	CXCL9
iCAF-CCL21	PRKG1
iCAF-CCL21	NR2F1
iCAF-CCL21	STPG2
iCAF-CCL21	THBS1
iCAF-CCL21	NPAS2
iCAF-CCL21	SNCAIP
iCAF-CCL21	PDE1C
iCAF-CCL21	PDE4D
iCAF-CCL21	PLPP1
iCAF-CCL21	LAMA2
iCAF-CCL21	RBM47
iCAF-CCL21	MEIS2
iCAF-CCL21	PTGER3
iCAF-CCL21	MAP1B
iCAF-CCL21	TMTC1
iCAF-CCL21	ROR1
iCAF-CCL21	NEBL
iCAF-CCL21	DHRS3
iCAF-CCL21	PLEKHH2
iCAF-CCL21	ADRA1B
iCAF-CCL21	PRICKLE1
iCAF-CCL21	SPARC
iCAF-CCL21	ZBTB16
iCAF-CCL21	SH3D19
iCAF-CCL21	CTGF

iCAF-CCL21	PGM5
iCAF-CCL21	RND3
iCAF-CCL21	STK32B
iCAF-CCL21	RP11-123O10.4
iCAF-CCL21	C10orf10
iCAF-CCL21	THY1
iCAF-CCL21	GAS7
iCAF-CCL21	KCNK13
iCAF-CCL21	RBMS3-AS2
iCAF-CCL21	KITLG
iCAF-CCL21	TMTC2
iCAF-CCL21	FBLN5
iCAF-CCL21	TGM2
iCAF-CCL21	LMCD1-AS1
iCAF-CCL21	DIRC3
iCAF-CCL21	FAM189A1
iCAF-CCL21	LAMA4
iCAF-CCL21	ARHGAP6
iCAF-CCL21	WISP1
iCAF-CCL21	THSD7A
iCAF-CCL21	PBX3
iCAF-CCL21	FHL1
iCAF-CCL21	EFEMP1
iCAF-CCL21	CEMIP
iCAF-CCL21	BIRC3
iCAF-CCL21	ANKRD33B
iCAF-CCL21	DENND2A
iCAF-CCL21	ERBB4
iCAF-CCL21	ERN1
iCAF-CCL21	HIVEP3
iCAF-CCL21	ERRFI1
iCAF-CCL21	PRSS23
iCAF-CCL21	CXCL10
iCAF-CCL21	RGL1
iCAF-CCL21	TWIST2
iCAF-CCL21	PLAGL1
iCAF-CCL21	FKBP5
iCAF-CCL21	MOXD1
iCAF-CCL21	DMD
iCAF-CCL21	ARHGAP22
iCAF-CCL21	KCNIP4
iCAF-CCL21	LMCD1
iCAF-CCL21	MIR100HG
iCAF-CCL21	CACNB4
iCAF-CCL21	ANTXR2
iCAF-CCL21	RBPJ
iCAF-CCL21	FGD5
iCAF-CCL21	L3MBTL4

iCAF-CCL21	PPFIA2
iCAF-CCL21	SAMD12
iCAF-CCL21	DAPK2
iCAF-CCL21	MAOB
iCAF-CCL21	F3
iCAF-CCL21	ST5
iCAF-CCL21	CCDC170
iCAF-CCL21	PDE10A
iCAF-CCL21	NID1
iCAF-CCL21	ZC4H2
iCAF-CCL21	CYR61
iCAF-CCL21	CCDC80
iCAF-CCL21	SLCO5A1
iCAF-CCL21	CYSLTR2
iCAF-CCL21	FAT4
iCAF-CCL21	TBC1D9
iCAF-CCL21	DAB1
iCAF-CCL21	SERPINE1
iCAF-CCL21	DOCK8
iCAF-CCL21	SRGN
iCAF-CCL21	PCSK5
iCAF-CCL21	LDLRAD3
iCAF-CCL21	RP11-356I2.4
iCAF-CCL21	FRMD6
iCAF-CCL21	TXNIP
iCAF-CCL21	WWTR1
iCAF-CCL21	ABCA6
iCAF-CCL21	GJA1
iCAF-CCL21	EYA2
iCAF-CCL21	ARHGAP44
iCAF-CCL21	NAV3
iCAF-CCL21	KIAA0825
iCAF-CCL21	ADAMTSL1
iCAF-CCL21	DKK2
iCAF-CCL21	CEP112
iCAF-CCL21	HMCN2
iCAF-CCL21	DPH6-AS1
iCAF-CCL21	CSTA
iCAF-CCL21	EPHB1
iCAF-CCL21	NUAK1
iCAF-CCL21	DPT
iCAF-CCL21	CBLB
iCAF-CCL21	BACE2
iCAF-CCL21	ATRNL1
iCAF-CCL21	C8orf4
iCAF-CCL21	COL5A3
iCAF-CCL21	RP11-705O24.1
iCAF-CCL21	RP11-69I8.3

iCAF-CCL21	FHL2
iCAF-CCL21	LINC01515
iCAF-CCL21	SLC7A2
iCAF-CCL21	SLC30A8
iCAF-CCL21	RP4-678D15.1
iCAF-CCL21	FCHO2
iCAF-CCL21	RP11-624C23.1
iCAF-CCL21	IRF8
iCAF-CCL21	MRVI1
iCAF-CCL21	UNC5B
iCAF-CCL21	ESRRG
iCAF-CCL21	SLC8A1-AS1
iCAF-CCL21	RP11-426C22.5
iCAF-CCL21	PDGFRA
iCAF-CCL21	HELLS
iCAF-CCL21	NRP1
iCAF-CCL21	NTRK3
iCAF-CCL21	GALNT18
iCAF-CCL21	AC144449.1
iCAF-CCL21	P3H2
iCAF-CCL21	KCNB2
iCAF-CCL21	HPGD
iCAF-CCL21	COL5A1
iCAF-CCL21	CADM2
iCAF-CCL21	RCAN2
iCAF-CCL21	LURAP1L-AS1
iCAF-CCL21	FLG-AS1
iCAF-CCL21	LINC01266
iCAF-CCL21	PLEKHA6
iCAF-CCL21	PKP4
iCAF-CCL21	CRISPLD2
iCAF-CCL21	ZNF208
iCAF-CCL21	RP5-965F6.2
iCAF-CCL21	OSBPL6
iCAF-CCL21	SH3RF3
iCAF-CCL21	DST
iCAF-CCL21	HMGCLL1
iCAF-CCL21	PAMR1
iCAF-CCL21	RP11-405A12.2
iCAF-CCL21	CYP7B1
iCAF-CCL21	RYR2
iCAF-CCL21	TMSB4X
iCAF-CCL21	HS6ST2
iCAF-CCL21	SYT1
iCAF-CCL21	ITGA9

iCAF-CCL21	SUGCT
iCAF-CCL21	MDM2
iCAF-CCL21	RP11-368L12.1
iCAF-CCL21	HSPA1A
iCAF-CCL21	AC007319.1
iCAF-CCL21	IGFBP5
iCAF-CCL21	ACKR1
iCAF-CCL21	BGN
iCAF-CCL21	CCBE1
iCAF-CCL21	NOVA1-AS1
iCAF-CCL21	VCAN
iCAF-CCL21	NID2
iCAF-CCL21	NTM
iCAF-CCL21	RP11-727A23.10
iCAF-CCL21	CHSY3
iCAF-CCL21	MNDA
iCAF-CCL21	JAG1
iCAF-CCL21	NCAM2
iCAF-CCL21	KCND3
iCAF-CCL21	PRR16
iCAF-CCL21	MIR325HG
iCAF-CCL21	EEPD1
iCAF-CCL21	FN1
iCAF-CCL21	SH3PXD2B
iCAF-CCL21	PCDH15
iCAF-CCL21	ZNF385D
iCAF-CCL21	FAM13C
iCAF-CCL21	LINC00968
iCAF-CCL21	ABCA9
iCAF-CCL21	THBS2
iCAF-CCL21	RP11-371F15.3
iCAF-CCL21	IL4I1
iCAF-CCL21	NOTCH3
iCAF-CCL21	TRHDE
iCAF-CCL21	MYL9
iCAF-CCL21	UBE2E2
iCAF-CCL21	MTHFD1L
iCAF-CCL21	ARHGAP20
iCAF-CCL21	SULF2
iCAF-CCL21	SLC27A6
iCAF-CCL21	VIT
iCAF-CCL21	INHBA
iCAF-CCL21	NTRK2
iCAF-CCL21	ZEB1-AS1
iCAF-CCL21	APLNR
iCAF-CCL21	RFX8

iCAF-CCL21	TMEM163
iCAF-CCL21	RHOBTB3
iCAF-CCL21	ITSN1
iCAF-CCL21	TNFRSF21
iCAF-CCL21	SHROOM4
iCAF-CCL21	CENPP
iCAF-CCL21	KIAA1462
cDC2_RTN1	SLC8A1
cDC2_RTN1	PLXDC2
cDC2_RTN1	RBM47
cDC2_RTN1	RTN1
cDC2_RTN1	AOAH
cDC2_RTN1	LYZ
cDC2_RTN1	C10orf11
cDC2_RTN1	TFEC
cDC2_RTN1	CHN2
cDC2_RTN1	PTPRE
cDC2_RTN1	CPVL
cDC2_RTN1	DPYD
cDC2_RTN1	PID1
cDC2_RTN1	SAMHD1
cDC2_RTN1	CIITA
cDC2_RTN1	TBXAS1
cDC2_RTN1	MS4A6A
cDC2_RTN1	RP11-426C22.5
cDC2_RTN1	FRMD4B
cDC2_RTN1	DOCK4
cDC2_RTN1	ITGAX
cDC2_RTN1	JAML
cDC2_RTN1	MRC1
cDC2_RTN1	CCSER1
cDC2_RTN1	CSF2RA
cDC2_RTN1	KYNU
cDC2_RTN1	RAB31
cDC2_RTN1	SLC9A9
cDC2_RTN1	MCTP1
cDC2_RTN1	FGL2
cDC2_RTN1	SLC8A1-AS1
cDC2_RTN1	LYN
cDC2_RTN1	HCK
cDC2_RTN1	CD86
cDC2_RTN1	TNFAIP2
cDC2_RTN1	FYB
cDC2_RTN1	FGD2
cDC2_RTN1	FAM49A
cDC2_RTN1	1-Mar
cDC2_RTN1	RNF144B
cDC2_RTN1	SLC1A3

cDC2_RTN1	HLA-DRA
cDC2_RTN1	IRAK3
cDC2_RTN1	EPSTI1
cDC2_RTN1	FKBP5
cDC2_RTN1	PLEK
cDC2_RTN1	TRPS1
cDC2_RTN1	HDAC9
cDC2_RTN1	DAPK1
cDC2_RTN1	RASSF4
cDC2_RTN1	PIK3R5
cDC2_RTN1	IL18
cDC2_RTN1	GAS7
cDC2_RTN1	DMXL2
cDC2_RTN1	CLEC7A
cDC2_RTN1	PSTPIP2
cDC2_RTN1	FPR3
cDC2_RTN1	ZNF804A
cDC2_RTN1	ENTPD1
cDC2_RTN1	RP11-202G18.1
cDC2_RTN1	LPCAT2
cDC2_RTN1	SLC2A9
cDC2_RTN1	EPB41L3
cDC2_RTN1	CLEC12A
cDC2_RTN1	ARHGAP22
cDC2_RTN1	ITPR2
cDC2_RTN1	MYO1F
cDC2_RTN1	SLCO3A1
cDC2_RTN1	ANKRD22
cDC2_RTN1	DOCK2
cDC2_RTN1	DOCK8
cDC2_RTN1	CD163
cDC2_RTN1	CAMK1D
cDC2_RTN1	RGL1
cDC2_RTN1	LRRK2
cDC2_RTN1	MSR1
cDC2_RTN1	CD74
cDC2_RTN1	UBE2E2
cDC2_RTN1	ABCA1
cDC2_RTN1	CXCL10
cDC2_RTN1	MYOF
cDC2_RTN1	KCNMA1
cDC2_RTN1	SLCO2B1
cDC2_RTN1	RP6-159A1.4
cDC2_RTN1	VOPP1
cDC2_RTN1	SAT1
cDC2_RTN1	ATP8B4
cDC2_RTN1	PRKCB
cDC2_RTN1	ARHGAP25

cDC2_RTN1	DSE
cDC2_RTN1	LCP2
cDC2_RTN1	ACSL1
cDC2_RTN1	ST8SIA4
cDC2_RTN1	HIVEP3
cDC2_RTN1	SYK
cDC2_RTN1	MAFB
cDC2_RTN1	RP11-212I21.2
cDC2_RTN1	P2RY14
cDC2_RTN1	FCN1
cDC2_RTN1	MS4A7
cDC2_RTN1	IL15
cDC2_RTN1	CPM
cDC2_RTN1	C1QB
cDC2_RTN1	APBB1IP
cDC2_RTN1	MIR181A1HG
cDC2_RTN1	CSF3R
cDC2_RTN1	RASGEF1B
cDC2_RTN1	ADAM28
cDC2_RTN1	MNDA
cDC2_RTN1	SAMSN1
cDC2_RTN1	LINC00278
cDC2_RTN1	CD38
cDC2_RTN1	MS4A4E
cDC2_RTN1	CLEC10A
cDC2_RTN1	ADAP2
cDC2_RTN1	FLI1
cDC2_RTN1	ELMO1
cDC2_RTN1	SULF2
cDC2_RTN1	P2RY6
cDC2_RTN1	ARHGAP26
cDC2_RTN1	CMKLR1
cDC2_RTN1	PLAGL1
cDC2_RTN1	ADGRE2
cDC2_RTN1	WARS
cDC2_RTN1	SFMBT2
cDC2_RTN1	TCF7L2
cDC2_RTN1	FNDC3B
cDC2_RTN1	SLAMF7
cDC2_RTN1	TRPM2
cDC2_RTN1	ITGA4
cDC2_RTN1	SRGN
cDC2_RTN1	PIK3AP1
cDC2_RTN1	ARHGAP18
cDC2_RTN1	PLA2G7
cDC2_RTN1	FCHO2
cDC2_RTN1	INPP5D

cDC2_RTN1	KCNK13
cDC2_RTN1	GBP1
cDC2_RTN1	WDFY4
cDC2_RTN1	C1QA
cDC2_RTN1	FAM46A
cDC2_RTN1	LDLRAD4
cDC2_RTN1	STARD13
cDC2_RTN1	TBC1D9
cDC2_RTN1	RAB20
cDC2_RTN1	NRP1
cDC2_RTN1	PBX3
cDC2_RTN1	VCAN
cDC2_RTN1	LRRK1
cDC2_RTN1	RBPJ
cDC2_RTN1	PHACTR2
cDC2_RTN1	HTR7
cDC2_RTN1	GPAT3
cDC2_RTN1	TCF4
cDC2_RTN1	EPB41L2
cDC2_RTN1	TGFBI
cDC2_RTN1	FLVCR2
cDC2_RTN1	CTSB
cDC2_RTN1	GRAMD1B
cDC2_RTN1	MB21D2
cDC2_RTN1	ARHGAP15
cDC2_RTN1	LY86
cDC2_RTN1	ME1
cDC2_RTN1	RP11-452H21.1
cDC2_RTN1	ZNF366
cDC2_RTN1	PDGFC
cDC2_RTN1	PTPRC
cDC2_RTN1	CD36
cDC2_RTN1	RAP1GAP2
cDC2_RTN1	MERTK
cDC2_RTN1	IFI44L
cDC2_RTN1	CEBPD
cDC2_RTN1	FLT3
cDC2_RTN1	APBA1
cDC2_RTN1	KMO
cDC2_RTN1	AXL
cDC2_RTN1	IL4I1
cDC2_RTN1	LGMN
cDC2_RTN1	ALCAM
cDC2_RTN1	HSPA1A
cDC2_RTN1	PHACTR1
cDC2_RTN1	SLA
cDC2_RTN1	VSIG4
cDC2_RTN1	TMSB4X

cDC2_RTN1	OSBPL3
cDC2_RTN1	CHST15
cDC2_RTN1	TXNIP
cDC2_RTN1	CCDC170
cDC2_RTN1	L3MBTL4
cDC2_RTN1	FTL
cDC2_RTN1	ZBTB46
cDC2_RTN1	FCHSD2
cDC2_RTN1	SIGLEC1
cDC2_RTN1	F13A1
cDC2_RTN1	TNFAIP3
cDC2_RTN1	SLC40A1
cDC2_RTN1	FCGR2A
cDC2_RTN1	SEPP1
cDC2_RTN1	MAN1A1
cDC2_RTN1	RUNX1
cDC2_RTN1	CCDC26
cDC2_RTN1	TANC2
cDC2_RTN1	CD163L1
cDC2_RTN1	GLUL
cDC2_RTN1	LILRB4
cDC2_RTN1	DNASE1L3
cDC2_RTN1	STON2
cDC2_RTN1	STAB1
cDC2_RTN1	C22orf34
cDC2_RTN1	FGD6
cDC2_RTN1	TMTC2
cDC2_RTN1	CYSLTR2
cDC2_RTN1	IKZF1
cDC2_RTN1	RP11-624C23.1
cDC2_RTN1	DYSF
cDC2_RTN1	CD83
cDC2_RTN1	CD14
cDC2_RTN1	NPL
cDC2_RTN1	FAM129A
cDC2_RTN1	CXCL9
cDC2_RTN1	PLD1
cDC2_RTN1	FCGR3A
cDC2_RTN1	IRF8
cDC2_RTN1	PPM1L
cDC2_RTN1	DTNA
cDC2_RTN1	GBE1
cDC2_RTN1	SIPA1L1
cDC2_RTN1	SRGAP1
cDC2_RTN1	RP11-638I2.8
cDC2_RTN1	FMNL2
cDC2_RTN1	MGLL
cDC2_RTN1	ELL2

cDC2_RTN1	IFI30
cDC2_RTN1	S100Z
cDC2_RTN1	PECAM1
cDC2_RTN1	KLHL6
cDC2_RTN1	PDK4
cDC2_RTN1	FRMD3
cDC2_RTN1	NCEH1
cDC2_RTN1	SEL1L3
cDC2_RTN1	TEC
cDC2_RTN1	TPRG1
cDC2_RTN1	FAM20A
cDC2_RTN1	SLC2A3
cDC2_RTN1	SASH1
cDC2_RTN1	SH3PXD2B
cDC2_RTN1	PAPSS2
cDC2_RTN1	ARHGAP24
cDC2_RTN1	MGAM
cDC2_RTN1	UBASH3B
cDC2_RTN1	FOS
cDC2_RTN1	RIN2
cDC2_RTN1	RP11-556E13.1
cDC2_RTN1	CENPP
cDC2_RTN1	LINC01146
cDC2_RTN1	GPRIN3
cDC2_RTN1	C4orf32
cDC2_RTN1	ITSN1
cDC2_RTN1	IL1B
cDC2_RTN1	MS4A4A
cDC2_RTN1	DLEU7
cDC2_RTN1	TNFAIP8
cDC2_RTN1	ACSM3
cDC2_RTN1	RGS2
cDC2_RTN1	ABCC3
cDC2_RTN1	SLC11A1
cDC2_RTN1	MRC2
cDC2_RTN1	SGK1
cDC2_RTN1	RP11-489O18.1
cDC2_RTN1	NEAT1
cDC2_RTN1	SPRED1
cDC2_RTN1	HMOX1
cDC2_RTN1	EEPD1
cDC2_RTN1	MIR4435-2HG
cDC2_RTN1	FTH1
cDC2_RTN1	SH3RF1
cDC2_RTN1	PPARG
cDC2_RTN1	LRMP

cDC2_RTN1	TRPC6
cDC2_RTN1	CACNA2D3
cDC2_RTN1	POU2F2
cDC2_RTN1	IER3
cDC2_RTN1	PFKFB4
cDC2_RTN1	NEGR1
cDC2_RTN1	RPS6KA2
cDC2_RTN1	PALD1
cDC2_RTN1	AC023590.1
cDC2_RTN1	CDCP1
cDC2_RTN1	EBI3
cDC2_RTN1	TMCC3
cDC2_RTN1	CCL3
cDC2_RTN1	ANPEP
cDC2_RTN1	BLNK
cDC2_RTN1	ANXA1
cDC2_RTN1	PLCG2
cDC2_RTN1	RP11-44K6.3
cDC2_RTN1	TENM4
cDC2_RTN1	LMO2
cDC2_RTN1	NHSL1
cDC2_RTN1	IGF1
cDC2_RTN1	HRH1
cDC2_RTN1	AC009784.3
cDC2_RTN1	RP11-142C4.6
cDC2_RTN1	OLR1
cDC2_RTN1	FAM107B
cDC2_RTN1	MEF2C
cDC2_RTN1	CTD-2341M24.1
cDC2_RTN1	IFIT2
cDC2_RTN1	GUCY1A3
cDC2_RTN1	TNS3
cDC2_RTN1	CSTA
cDC2_RTN1	SLC24A4
cDC2_RTN1	PRDM1
cDC2_RTN1	AIM2
cDC2_RTN1	IL2RA
cDC2_RTN1	P2RX7
cDC2_RTN1	AC026188.1
cDC2_RTN1	MIR4300HG
cDC2_RTN1	CTD-2282P23.2
cDC2_RTN1	TMSB10
cDC2_RTN1	PLCB1
cDC2_RTN1	OSBPL6
cDC2_RTN1	FRY
cDC2_RTN1	BTBD11

cDC2_RTN1	BASP1
cDC2_RTN1	BCAT1
cDC2_RTN1	HSPA6
cDC2_RTN1	GFRA2
cDC2_RTN1	MMP9
cDC2_RTN1	COLEC12
cDC2_RTN1	DIRC3
cDC2_RTN1	RASAL2
cDC2_RTN1	CR1
cDC2_RTN1	EAF2
cDC2_RTN1	MMS22L
cDC2_RTN1	CCL8
cDC2_RTN1	HGF
cDC2_RTN1	PLA2G4C
cDC2_RTN1	ALDH1A2
cDC2_RTN1	TGFA
cDC2_RTN1	PCSK5
cDC2_RTN1	CYSLTR1
cDC2_RTN1	VCAM1
cDC2_RTN1	ANTXR2
cDC2_RTN1	CCL4L2
cDC2_RTN1	CCL4
cDC2_RTN1	IL1R1
cDC2_RTN1	ID2
cDC2_RTN1	APOC1
cDC2_RTN1	DNAJB1
cDC2_RTN1	ADAM19
cDC2_RTN1	CD93
cDC2_RTN1	DPH6-AS1
cDC2_RTN1	ARNTL2
cDC2_RTN1	DKK2
cDC2_RTN1	STPG2
cDC2_RTN1	RNLS
cDC2_RTN1	PIP5K1B
cDC2_RTN1	PARM1
cDC2_RTN1	HDX
cDC2_RTN1	CXCL12
cDC2_RTN1	LINC00996
B_CD74	BANK1
B_CD74	MS4A1
B_CD74	CD74
B_CD74	HLA-DRA
B_CD74	FCRL1
B_CD74	KHDRBS2
B_CD74	BCL11A
B_CD74	PAX5
B_CD74	COL19A1
B_CD74	LTB
B_CD74	TMSB4X

B_CD74	RP11-624C23.1
B_CD74	ADAM28
B_CD74	EBF1
B_CD74	BLK
B_CD74	RALGPS2
B_CD74	PLEKHG1
B_CD74	CCSER1
B_CD74	STEAP1B
B_CD74	LINC00926
B_CD74	BACH2
B_CD74	FAM129C
B_CD74	FCER2
B_CD74	HDAC9
B_CD74	MEF2C
B_CD74	WDFY4
B_CD74	CD22
B_CD74	NEDD9
B_CD74	1-Mar
B_CD74	FCHSD2
B_CD74	ADAM19
B_CD74	CDK14
B_CD74	BIRC3
B_CD74	PLCG2
B_CD74	BLNK
B_CD74	PRKCB
B_CD74	CR2
B_CD74	ARHGAP24
B_CD74	CD83
B_CD74	EAF2
B_CD74	RHOH
B_CD74	KMO
B_CD74	AFF3
B_CD74	SEL1L3
B_CD74	POU2F2
B_CD74	SETBP1
B_CD74	RP11-498M5.2
B_CD74	LY86
B_CD74	PIK3AP1
B_CD74	LYN
B_CD74	CR1
B_CD74	IKZF3
B_CD74	SYK
B_CD74	KCNQ5
B_CD74	TNFAIP8
B_CD74	CAMK1D
B_CD74	RPS21
B_CD74	CIITA

B_CD74	FCRL2
B_CD74	IGLC2
B_CD74	MACROD2
B_CD74	ALOX5
B_CD74	INPP5D
B_CD74	PLAC8
B_CD74	RPS18
B_CD74	TMEM156
B_CD74	ARHGAP15
B_CD74	PTPRK
B_CD74	C12orf42
B_CD74	KYNU
B_CD74	MCTP2
B_CD74	ITGA4
B_CD74	IGHM
B_CD74	RP11-142C4.6
B_CD74	C1orf186
B_CD74	IRF8
B_CD74	IKZF1
B_CD74	PIP5K1B
B_CD74	VAV3
B_CD74	FAM65B
B_CD74	CTD-2215E18.1
B_CD74	LY86-AS1
B_CD74	KCNH8
B_CD74	CARD11
B_CD74	DOCK2
B_CD74	MEF2C-AS1
B_CD74	PPP1R16B
B_CD74	FGD2
B_CD74	FCRL3
B_CD74	PTPRC
B_CD74	DOCK8
B_CD74	AC013463.2
B_CD74	SLC38A11
B_CD74	TCF4
B_CD74	LRMP
B_CD74	TXNIP
B_CD74	IGKC
B_CD74	ENTPD1
B_CD74	RFTN1
B_CD74	KLHL14
B_CD74	COBLL1
B_CD74	BASP1
B_CD74	ELMO1
B_CD74	LRRK2
B_CD74	LINC01572

B_CD74	RASGRP3
B_CD74	FLI1
B_CD74	ST8SIA4
B_CD74	SIPA1L1
B_CD74	CYSLTR1
B_CD74	ACTB
B_CD74	RP11-96H19.1
B_CD74	XXbac-BPG154L12.5
B_CD74	PTMA
B_CD74	POU2AF1
B_CD74	ATP5E
B_CD74	APBB1IP
B_CD74	MT-ND1
B_CD74	TMSB10
B_CD74	FCRL5
B_CD74	FAM49A
B_CD74	ALCAM
B_CD74	AIM2
B_CD74	UBE2E2
B_CD74	NETO1
B_CD74	FAM107B
B_CD74	ACSM3
B_CD74	GPM6A
B_CD74	MT-ND2
B_CD74	PCED1B
B_CD74	TBC1D9
B_CD74	ST6GALNAC3
B_CD74	P2RY14
B_CD74	PBX3
B_CD74	SCN3A
B_CD74	ABLIM1
B_CD74	AC093818.1
B_CD74	RP11-131L23.1
B_CD74	MT-ND3
B_CD74	RP6-159A1.4
B_CD74	SAMD12
B_CD74	THRB
B_CD74	SYN3
B_CD74	CTD-2509G16.5
B_CD74	ITPR2
B_CD74	L3MBTL4
B_CD74	IGLL5
B_CD74	C22orf34
B_CD74	PHACTR1

B_CD74	MT-ATP6
B_CD74	KLF12
B_CD74	RUNX1
B_CD74	HIST1H4C
B_CD74	RP11-265P11.1
B_CD74	ANKRD33B
B_CD74	PDE7B
B_CD74	ARHGAP25
B_CD74	CLNK
B_CD74	MT-CO3
B_CD74	VOPP1
B_CD74	PDK1
B_CD74	STK33
B_CD74	RGS7
B_CD74	MTSS1
B_CD74	CHL1
B_CD74	EPSTI1
B_CD74	ID3
B_CD74	AC007879.5
B_CD74	XYLT1
B_CD74	ADGRG5
B_CD74	SAMSN1
B_CD74	IFI44L
B_CD74	XACT
B_CD74	DIRC3
B_CD74	RP11-356N1.2
B_CD74	SLCO5A1
B_CD74	MACC1
B_CD74	FTH1
B_CD74	COL4A4
B_CD74	CTD-2341M24.1
B_CD74	ZBTB16
B_CD74	DDIT4
B_CD74	CHST15
B_CD74	MT-CYB
B_CD74	SYT1
B_CD74	PARM1
B_CD74	TMEM163
B_CD74	EPB41L2
